# Reassessing Domain Architecture Evolution of Metazoan Proteins: Major Impact of Gene Prediction Errors

**DOI:** 10.3390/genes2030449

**Published:** 2011-07-13

**Authors:** Alinda Nagy, György Szláma, Eszter Szarka, Mária Trexler, László Bányai, László Patthy

**Affiliations:** Institute of Enzymology, Biological Research Center, Hungarian Academy of Sciences, H-1113 Budapest, Hungary; E-Mails: nagya@enzim.hu (A.N.); szlama@enzim.hu (G.S.); szarka@enzim.hu (E.S.); trexler@enzim.hu (M.T.); banyai@enzim.hu (L.B.)

**Keywords:** domain architecture, evolution of domain architecture, gene prediction error, multidomain protein, orthologs, quality control of gene prediction

## Abstract

In view of the fact that appearance of novel protein domain architectures (DA) is closely associated with biological innovations, there is a growing interest in the genome-scale reconstruction of the evolutionary history of the domain architectures of multidomain proteins. In such analyses, however, it is usually ignored that a significant proportion of Metazoan sequences analyzed is mispredicted and that this may seriously affect the validity of the conclusions. To estimate the contribution of errors in gene prediction to differences in DA of predicted proteins, we have used the high quality manually curated UniProtKB/Swiss-Prot database as a reference. For genome-scale analysis of domain architectures of predicted proteins we focused on RefSeq, EnsEMBL and NCBI's GNOMON predicted sequences of Metazoan species with completely sequenced genomes. Comparison of the DA of UniProtKB/Swiss-Prot sequences of worm, fly, zebrafish, frog, chick, mouse, rat and orangutan with those of human Swiss-Prot entries have identified relatively few cases where orthologs had different DA, although the percentage with different DA increased with evolutionary distance. In contrast with this, comparison of the DA of human, orangutan, rat, mouse, chicken, frog, zebrafish, worm and fly RefSeq, EnsEMBL and NCBI's GNOMON predicted protein sequences with those of the corresponding/orthologous human Swiss-Prot entries identified a significantly higher proportion of domain architecture differences than in the case of the comparison of Swiss-Prot entries. Analysis of RefSeq, EnsEMBL and NCBI's GNOMON predicted protein sequences with DAs different from those of their Swiss-Prot orthologs confirmed that the higher rate of domain architecture differences is due to errors in gene prediction, the majority of which could be corrected with our FixPred protocol. We have also demonstrated that contamination of databases with incomplete, abnormal or mispredicted sequences introduces a bias in DA differences in as much as it increases the proportion of terminal over internal DA differences. Here we have shown that in the case of RefSeq, EnsEMBL and NCBI's GNOMON predicted protein sequences of Metazoan species, the contribution of gene prediction errors to domain architecture differences of orthologs is comparable to or greater than those due to true gene rearrangements. We have also demonstrated that domain architecture comparison may serve as a useful tool for the quality control of gene predictions and may thus guide the correction of sequence errors. Our findings caution that earlier genome-scale studies based on comparison of predicted (frequently mispredicted) protein sequences may have led to some erroneous conclusions about the evolution of novel domain architectures of multidomain proteins. A reassessment of the DA evolution of orthologous and paralogous proteins is presented in an accompanying paper [1].

## 1. Introduction

In view of the fact that appearance of novel protein domain architectures (DA) is closely associated with biological innovations [2,3] there is a growing interest in the genome-scale analysis of the evolutionary history of the domain architectures of multidomain proteins and the contribution of different evolutionary mechanisms to changes in domain architectures.

Reliable reconstruction of the evolutionary history of the DA of multidomain proteins requires that: (1) the protein sequences compared are valid, correct and complete; (2) DAs are determined accurately and their differences are detected with high specificity and sensitivity; (3) the evolutionary relationship of multidomain proteins compared is correctly defined. A survey of recent papers describing analyses of the evolutionary history of the DA of proteins, however, suggests that problems with each of these points may have had a strong impact on the validity of the conclusions.

### 1.1. Requirement 1: The Protein Sequences Compared are Valid, Correct and Complete

A general problem of studies on DA evolution is that true change of DA (at the genome level) may be confused with change of DA only at the transcript level, due to alternative splicing. As discussed in the present manuscript this type of problem is sometimes encountered even in the case of high-quality, manually curated Swiss-Prot section of UniProtKB; different isoforms (with different DA) are presented for orthologous genes with similar genomic structure.

Although the Swiss-Prot section of UniProtKB contains only a few non-valid, incomplete or erroneous sequences, the TrEMBL section of UniProtKB is heavily contaminated with N-terminally or C-terminally truncated and chimeric sequences [4]. In view of this fact, data obtained by analyses of UniProtKB datasets containing both the Swiss-Prot and TrEMBL section of UniProtKB may be biased in favor of differences at the N- and C-termini of proteins. It is noteworthy in this respect that—based on analyses of whole UniProtKB (SwissProt plusTrEMBL) sets of proteins—Weiner *et al.* [5] concluded that domain losses and duplications were more frequent at the ends of proteins. This finding led the authors to conclude that the genetic mechanism leading to DA changes acts predominantly on sequence termini and that modular evolution of proteins is dominated by two major types of events: fusion, on the one hand, and deletion and fission on the other.

To estimate the contribution of sequence errors to differences in DA, in the present work we have compared data obtained on the high quality, manually curated Swiss-Prot database with data obtained on TrEMBL sequences of Metazoa. Our analyses have confirmed that DA differences due to errors of TrEMBL sequences may significantly exceed the rate of true DA changes.

In the case of genome-scale analyses of DA changes, the majority of protein sequences analyzed is predicted: the accuracy of the predicted sequences depends on the type of genome and the performance of protocols used for the identification of protein-coding genes in genomic sequences. Protein-coding genes encoded by intron-poor genomes are usually predicted with great specificity and sensitivity. However, correct prediction of the genomic structure of the protein-coding genes of higher eukaryotes with intron-rich genomes is still a very difficult task. Recent analyses have shown that the exact genomic structure of protein-coding genes of higher eukaryotes is correctly predicted for only about 60% of the genes [6]. Since many types of misprediction lead to terminal truncation and fusion of proteins [4] the high rate of misprediction is expected to have a major impact on conclusions drawn from genome-scale DA comparisons of Metazoan proteins.

It must also be pointed out that computational gene prediction introduces a strong positional bias in the distribution of errors in as much as the initial and terminal exons of genes are predicted with significantly lower accuracy than internal exons [7]. In terms of DA this means that DA differences due to misprediction are more likely to be observed at the N-terminal end and the C-terminal end than internally.

Although many authors realize that some of the DA differences result from errors of gene prediction, the contribution of this to DA differences has not been explored. To estimate the contribution of errors of gene prediction to differences in DA in the present work we have compared data obtained on the high quality, manually curated Swiss-Prot database with data obtained on databases containing less reliable, predicted sequences (e.g., RefSeq, EnsEMBL and NCBI's GNOMON predicted protein sequences) of Metazoa.

Our analyses have shown that DA differences due to errors in gene prediction may significantly exceed the rate of true DA change; therefore domain architecture comparison may serve as a tool for the quality control of gene predictions and may guide the correction of sequence errors. We have shown that application of this approach significantly improves the quality of gene predictions and promotes more reliable identification of true cases of domain architecture changes.

Our finding, that errors in gene prediction significantly distort the patterns of DA evolution, cautions that earlier studies based on comparison of predicted (frequently mispredicted) protein sequences may have led to some erroneous conclusions about the evolution of novel domain architectures of multidomain proteins. The influence of gene prediction errors on DA evolution of orthologous and paralogous proteins is discussed in an accompanying paper [1].

### 1.2. Requirement 2: DAs are Determined Accurately and Their Differences are Detected with High Specificity and Sensitivity

The choice of protocol for domain identification may have a strong influence on the specificity and sensitivity of domain architecture comparison. Since many domain-types defined by the Pfam database [8,9] or the CDD database [10,11] are not represented in the CATH database [12,13] or the SCOP database [14,15] the use of CATH or SCOP for domain identification provides a lower resolution of DA comparison than others: DA differences involving Pfam A or CDD domains of unknown 3D structure (and thus missing from SCOP and CATH) will remain undetected. Studies that used CATH or SCOP for definition of DAs [16,17] are thus expected to underestimate DA differences more than the ones that use Pfam A defined by Pfam or CDD (the latter provide a better coverage of proteins). To get a higher resolution of DA differences, in the present study we have used a CDD/Pfam A-based procedure to determine DAs of proteins.

The definition of ‘domain architecture’ of proteins also has a strong influence on the conclusions that can be drawn from comparison of proteins. A survey of the literature reveals that, although the majority of authors define DA as the linear sequence of constituent domains from the N-terminus to the C-terminus [2,18,19], more relaxed and more stringent definitions are also used. Some studies analyzed ‘domain-combinations’, ‘set of domains’ (irrespective of the order of domains) to get insights into evolution of multidomain proteins [20,21], others analyzed local (domain-pair) architectures to get an insight into changes of global domain architectures [22,23]. Since the same domain combinations or local domain-combinations may evolve independently [24,25] the use of these definitions underestimates DA changes. On the other hand, sometimes the distance (length of ‘linkers’) separating consecutive domains was used to distinguish architectures in which two domains are adjacent (e.g., less than 30 residues between domains) from those that are separated by a longer segment [16]. In the case of proteins with longer disordered linker regions this definition may judge similar architectures to be different and may thus overestimate DA changes. In the present work we used the ‘standard’ definition of domain architecture: the linear sequence of constituent domains.

In some analyses, uninterrupted tandem repeats of the same domain-type are collapsed into a single pseudo-domain, therefore these analyses do not detect tandem duplication of a domain or deletion of a tandem duplicated domain [24,26]. To get a better view of the contribution of different types of DA changes, in the present work we defined domain architecture as the linear sequence of constituent domains, including tandem copies of the same domain-type.

Although it is clear that the choice of protocols and choice of cut-off values for domain identification have a major influence on DA comparison, very little is known about the sensitivity and specificity of the procedures used to detect differences in DA in the various studies. In the absence of this information it is not known to what extent the various approaches used in the different studies overestimate or underestimate DA differences.

To overcome this problem, in the present work we optimized our DA comparison protocol using the high quality manually curated Swiss-Prot database as a benchmark. False positive rate and specificity of detection of DA differences were determined by comparing orthologous Swiss-Prot entries known to have identical domain architectures. False negative rate and sensitivity of detection of DA differences were determined using datasets of orthologous Swiss-Prot entries with artificially altered domain architectures.

### 1.3. Requirement 3: The Evolutionary Relationship between the Multidomain Proteins Compared is Correctly Defined

To estimate the contribution of different types of events to changes in domain architecture one has to correctly define the evolutionary relationship between the homologous multidomain proteins that are compared.

Establishing the evolutionary relationship of multidoman proteins, however, is not trivial. First, since in many cases not all parts of two homologous multidomain proteins have the same evolutionary history, the usual terms for homology (orthology, paralogy) of proteins may not apply. Considering these problems, it has been suggested that the use of the concept of orthology is applicable only at the level of domains rather than at the level of proteins, except for proteins with identical domain architectures [27,28]. The exact evolutionary relationship of homologous multidomain proteins may be defined only through the analysis of the evolutionary histories of their constituent domains [29], but no automatic procedure exists that can perform such analyses on a mass-scale. As a consequence of these problems it is generally accepted that the procedures used for orthology or paralogy group construction and construction of sequence-based gene trees are more likely to misassign multidomain proteins than single domain proteins [30–32].

Despite these caveats, some authors analyzing DA changes have relied on trees determined for entire multidomain proteins [33,34], but most studies have circumvented the problem of sequence-based phylogeny of multidomain proteins by using phylogenies based on similarities of domain architectures [21,24,26,35, 36]. The problem with this approach, however, is that it may distort true evolutionary relationships: distantly related proteins with more similar domain architectures may appear to be closely related; closely related proteins with less similar architectures may appear to be distantly related and tends to underestimate the number of DA changes. It is noteworthy in this respect that it is well established that the same DA may evolve independently [25].

In the present work we have used sequence-based phylogeny of entire multidomain proteins and checked the reliability of orthology/paralogy assignments on representative samples of correctly annotated Swiss-Prot entries of multidomain proteins. Our results have shown that standard procedures used for establishing orthology are quite accurate even for multidomain proteins, but are much less reliable in defining groups of paralogs. The latter problem will be discussed in an accompanying paper [37].

## 2. Results and Discussion

### 2.1. Simulation of the Impact of Sequence Errors and Gene Prediction Errors on DA Differences of Orthologous Proteins

Comparison of DAs of Swiss-Prot proteins with those of artificially altered orthologs mimicking gene prediction errors (generated as described in the Experimental Section) revealed that artificial chimeras mimicking fusion of neighboring genes were detected as differing from their parents/orthologs in domain architecture in the majority (98%) of the cases. In other words, the present procedure is quite sensitive for the detection of fusion of tandem genes that appear as DA changes of the terminal type.

In the case of artificial (terminal or internal) deletions of 100 amino acid residues (simulating gene prediction errors that miss some true exons but do not shift the reading frame) only 12% of the sequences are detected as having altered DA. The explanation for this relatively weak effect on DA, is that the deletion of 100 residues did not always affect a Pfam A domain and, even if it did, it rarely removed an entire Pfam A domain and the truncated Pfam A domain was still detected.

In the case of artificial (terminal or internal) additions of 100 residues taken from other proteins (mimicking the erroneous inclusion of true protein-coding exons) a high proportion (75%) of the artificial sequences had domain architectures different from those of their parents: the introduced fragment (that may have contained a Pfam A domain or a fragment of a Pfam A domain) was detected with our protocol.

It seems likely that this asymmetry in the effect of erroneous omission or erroneous addition of a true exon (that does not cause reading frame shift) on DA has a significant impact on domain architecture comparisons. Since very few genes contain nested genes [38–40] that could erroneously contribute true Pfam A domains internally, whereas gene prediction may erroneously include exons of neighboring genes it is expected that misprediction will introduce a bias in favor of terminal over internal DA differences.

Addition of 100 residue-long random amino acid sequences at the termini of proteins (mimicking gene prediction errors in which terminal false exons are included) had no effect on the domain architectures, whereas internal insertion of such sequences (mimicking inclusion of false internal exons) resulted in change of domain architecture in a relatively high proportion (25%) of the cases. The explanation for this observation is that internal insertion of random sequences have sometimes split Pfam A domains and this led either to failure in domain identification or virtual ‘duplication’ of the split domain. Nevertheless, erroneous inclusion of false exons will not introduce a positional bias in DA change, since domains may be affected irrespective of their internal or terminal position.

So far we discussed only gene prediction errors that do not disrupt the reading frame. Mispredictions that result in reading frame-shift lead to truncation downstream of the point of such misprediction; irrespective of their position within the gene they lead to C-terminal truncation and will appear as C-terminal DA change.

Similarly, indel-type of errors of cDNA sequences resulting from cloning or sequencing errors may cause reading frame shift (if the indel involves 3n + 1 or 3n + 2 nucleotides) and this will lead to truncation downstream of the point of such an error; irrespective of their position within the gene they lead to C-terminal truncation and might appear as C-terminal DA change. For example, a single base ‘deletion’ caused by such an error [41] resulted in the apparent truncation of the Lgl1 protein (now known as cysteine-rich secretory protein LCCL domain containing 2 protein) and the apparent “loss” of two C-terminal LCCL domains that were shown to be present in the protein encoded by the correct cDNA sequence [42].

Finally, it should be pointed out that current computational gene prediction tools introduce a strong positional bias in the distribution of errors, in as much as the initial (“N-terminal”) exons are predicted with the lowest accuracy, terminal (“C-terminal”) exons of genes are predicted with somewhat greater accuracy, whereas internal exons are predicted with high accuracy [6]. In terms of DA this means that DA differences due to misprediction are much more likely to be observed at the N-terminal end and the C-terminal end than internally.

### 2.2. Comparison of the DA of Human Swiss-Prot Proteins with Orthologous Metazoan Swiss-Prot Proteins

The manually curated Swiss-Prot section of UniProtKB is the gold standard of protein databases therefore we have used Swiss-Prot as the benchmark to define the rate of domain architecture changes during evolution of orthologous proteins since this analysis is unlikely to be affected by sequence errors. An obvious limitation of the comparison of SwissProt entries, however, is that in the case of most species only a fraction of their proteomes is represented in this database. To permit statistically significant analyses, we have selected only Metazoa species with more than 1000 entries in Swiss-Prot: *Homo sapiens, Pongo abelii, Mus musculus, Rattus norvegicus, Gallus gallus, Xenopus tropicalis, Danio rerio, Caenorhabditis elegans* and *Drosophila melanogaster*.

The species thus selected represent different evolutionary groups of Metazoa and include species that diverged relatively recently (e.g., *Pongo-Homo*) as well as protostome species (worm and fly) that diverged from deuterostomes ∼1000 Mya. Comparison of proteins from different species permitted the analysis of the influence of evolutionary distance on domain architecture of orthologous proteins. (Divergence times of the species analyzed in this paper were taken from Table S1 [43,44].

Comparison of the DA of orthologous UniProtKB/Swiss-Prot sequences of worm, fly and several vertebrate species has identified few cases where orthologs apparently had different DA although the percentage of orthologs with different DA increased with evolutionary distance of the species compared: *Homo-Pongo*: 0.3%; *Homo-Mus*: 1.1%; *Homo-Gallus*: 3.00%; *Homo-Xenopus*: 0.9%; *Homo-Danio*: 2.1%; *Homo-Drosophila*: 4.8%; *Homo-Caenorhabditis*: 5.9% (Table 1/A). These results suggest that the rate of DA alteration is very low in the case of orthologs: apparently, the DA of ∼5% of the orthologs is changed over 1000 My.

Orthologous Swiss-Prot proteins with different domain architectures were subjected to in-depth analyses to decide whether deviation in domain architecture reflects errors in DA comparison (false positive) or the protein architectures are truly different due to some type of sequence error, alternative splicing or evolutionary change in domain architecture at the gene level. These analyses have shown that a small proportion (0.1% of orthologous pairs) is false positive, consistent with the fact that the specificity of the protocol used to determine DA differences is 0.985 (see Experimental Section).

**Table 1:** 

**Table 1/A** Proportion of Swiss-Prot sequences of Metazoan species that differ in DA from their human Swiss-Prot ortholog.				

Species*	**Database**	**Orthologous pairs**	**Orthologous Pairs with Different DA**	**Percent of Pairs with Different DA**
*Pongo abelii*	Swiss-Prot	2156	6	0,3
*Mus musculus*	Swiss-Prot	14522	167	1,1
*Gallus gallus*	Swiss-Prot	1799	54	3
*Xenopus tropicalis*	Swiss-Prot	1371	13	0,9
*Danio rerio*	Swiss-Prot	**1961**	42	2,1
*Drosophila melanogaster*	Swiss-Prot	**1038**	50	4,8
*Caenorhabditis elegans*	Swiss-Prot	852	50	5,9

*The species are listed in the order of increasing evolutionary distance from *Homo sapiens*.				

**Table 1/B** Proportion of TrEMBL sequences of Metazoan species that differ in DA from their human Swiss-Prot equivalent/ortholog.				

Species*	**Database**	**Pairs**	**Pairs with Different DA**	**Percent of Pairs with Different DA**
*Homo sapiens*	TrEMBL	13699	659	4,81
*Mus musculus*	TrEMBL	12196	489	4,01
*Gallus gallus*	TrEMBL	7055	312	4,42
*Xenopus tropicalis*	TrEMBL	6945	324	4,67
*Danio rerio*	TrEMBL	9001	450	5,00
*Drosophila melanogaster*	TrEMBL	5010	473	9,44
*Caenorhabditis elegans*	TrEMBL	4115	507	12,32

*The species are listed in the order of increasing evolutionary distance from *Homo sapiens*.				

**Table 1/C** Proportion of RefSeq sequences of Metazoan species that differ in DA from their human Swiss-Prot equivalent/ortholog.				

Species*	**Database**	**Pairs**	**Pairs with Different DA**	**Percent of Pairs with Different DA**
*Homo sapiens*	RefSeq	18245	70	0,38
*Mus musculus*	RefSeq	16490	247	1,50
*Gallus gallus*	RefSeq	11584	442	3,82
*Xenopus tropicalis*	RefSeq	7264	224	3,08
*Danio rerio*	RefSeq	1**2043**	571	4,74
*Drosophila melanogaster*	RefSeq	4951	496	10,02
*Caenorhabditis elegans*	RefSeq	5267	575	10,92

*The species are listed in the order of increasing evolutionary distance from *Homo sapiens*.				

**Table 1/D** Proportion of NCBI's GNOMON predicted sequences of Metazoan species that differ in DA from their human Swiss-Prot equivalent/ortholog.				

Species*	**Database**	**Pairs**	**Pairs with different DA**	**Percent of pairs with different DA**
*Homo sapiens*	NCBI	1355	107	7,90
*Mus musculus*	NCBI	2223	165	7,42
*Gallus gallus*	NCBI	11584	404	3,49
*Danio rerio*	NCBI	5630	557	9,89
*Drosophila pseudoobscura*	NCBI	5929	493	8,32
*Caenorhabditis briggsae*	NCBI	5130	612	11,93

*The species are listed in the order of increasing evolutionary distance from *Homo sapiens*.				

**Table 1/E** Proportion of EnsEMBL sequences of Metazoan species that differ in DA from their human Swiss-Prot equivalent/ortholog.				

Species*	**Database**	**Pairs**	**Pairs with different DA**	**Percent of pairs with different DA**
*Homo sapiens*	EnsEMBL	10915	119	1,09
*Mus musculus*	EnsEMBL	16508	259	1,57
*Gallus gallus*	EnsEMBL	11857	462	3,90
*Xenopus tropicalis*	EnsEMBL	11198	645	5,76
*Danio rerio*	EnsEMBL	11596	379	3,27
*Drosophila melanogaster*	EnsEMBL	6072	547	9,01
*Caenorhabditis elegans*	EnsEMBL	5130	553	10,78

*The species are listed in the order of increasing evolutionary distance from *Homo sapiens*.				

**Table 1/F** Proportion of RefSeq sequences of Metazoan species that differ in DA from their human RefSeq ortholog.				

Species*	**Pairs**	**Pairs with different DA**	**Percent of pairs with different DA**
*Mus musculus*	17207	530	3,08
*Gallus gallus*	11729	760	6,48
*Xenopus tropicalis*	7292	394	5,40
*Danio rerio*	12155	992	8,16
*Drosophila melanogaster*	4985	849	17,03
*Caenorhabditis elegans*	5330	835	15,67

*The species are listed in the order of increasing evolutionary distance from *Homo sapiens*.				

**Table 1/G** Proportion of NCBI's GNOMON predicted sequences of Metazoan species that differ in DA from their human RefSeq equivalent/ortholog.				

Species*	**Pairs**	**Pairs with different DA**	**Percent of pairs with different DA**
*Mus musculus*	2337	217	9,29
*Gallus gallus*	8274	662	8,00
*Danio rerio*	5691	809	14,22
*Drosophila pseudoobscura*	6014	859	14,28
*Caenorhabditis briggsae*	5195	863	16,61

* The species are listed in the order of increasing evolutionary distance from *Homo sapiens*.

Orthologous Swiss-Prot proteins with truly different DAs were subjected to additional analyses to assign them to one of the remaining categories. As described in the Experimental Section, orthologous protein sequences that differed in domain architecture in species A and B were used as queries to search the appropriate sections of various sequence databases (e.g., UniProtKB/TrEMBL, NCBI's protein and nucleic acid databases, EST databases) to decide whether the other species has a sequence that has the same domain architecture as the query. If such sequences were found it was concluded that the domain architecture difference observed in the case of Swiss-Prot entries is due either to a sequence error or to alternative splicing.

Our analyses revealed that some DA differences reflect alternative splicing, *i.e.*, the Swiss-Prot database presents different isoforms of the orthologs of different species, although their isoform pattern is similar. A typical example is agrin, where different splice forms (with different DAs) are given for different vertebrate species (AGRIN_HUMAN *vs.* AGRIN_MOUSE or AGRIN_RAT, Figure S1) although it is known that these differences are not species specific [45].

Another source of DA deviation of orthologous proteins is that one or both Swiss-Prot entries are not full-length proteins (note that we have shown previously that even Swiss-Prot database is contaminated with fragment or abnormal sequences [3]. For example, the DA of DCLK1_RAT differs significantly from those of DCLK1_MOUSE and DCLK1_HUMAN (TreeFam tree TF318770); whereas the latter contain two N-terminal DCX domains and a C-terminal Pkinase domain, the rat sequence lacks DCX domains. A full-length sequence predicted by the FixPred protocol confirmed that the DA of DCLK1_RAT_corrected is identical with those of DCLK1_MOUSE and DCLK_HUMAN (Figure S2.) Note that the N-terminal truncation of DCLK1_RAT appears as a DA change of the N-terminal-type.

There are cases where differences in DA of orthologous Swiss-Prot entries are due to misprediction. We illustrate this point with the case of SYWM_CAEEL (TreeFam tree TF314321). SYWM_CAEEL differs from SYWM_HUMAN (and other mitochondrial Tryptophanyl-tRNA synthetases from slime mold, yeast to mammals) in as much as it contains, in addition to the common tRNA-synt_1b domain, an extra N-terminal Pex2_Pex12 domain. This extra N-terminal region is most closely related to peroxisome biogenesis factor 10 of various species, raising the possibility that in *C. elegans* a mitochondrial protein has been fused to a peroxisomal protein. Reexamination of the genomic region encoding this protein, however, indicates that this “fusion” is the result of an error in gene prediction. EST BJ806113 of *Caenorhabditis elegans* and EST DR782673 of *Caenorhabditis remanei* support the existence of separate genes for a SYWM_CAEEL protein and a peroxisome biogenesis factor 10 ortholog, permitting the correction of the sequence of SYWM_CAEEL and the separation of the worm ortholog of PEX10_HUMAN (which we named as PEX10_CAEEL protein in Figure S3 a, b and c) using the FixPred protocol. Note that from the perspective of both PEX10 and SYWM the DA change in worm/human comparison appears as terminal change.

If the previous steps failed to identify sequences or isoforms that eliminated DA deviation we asked whether this is due to a change in gene structure. To achieve this, the appropriate genomic regions of the orthologous proteins were subjected to gene prediction to decide whether the domain(s) distinguishing the orthologs are encoded in both genomes or not. If this analysis confirmed that the altered domain architecture is due to a change in gene structure (a change in splicing, deletion/insertion/duplication of genomic regions, *etc.*) then it was concluded that an evolutionary change has occurred at the gene level that changed the domain architecture of the encoded protein(s).

An illustrative example for deletion of a unique internal domain comes from analysis of orthologs of the human tyrosine kinase MUSK (TreeFam tree TF106465). MUSK_HUMAN, MUSK_MOUSE, MUSK-RAT all contain three N-terminal I-set domains, an Fz domain and a C-terminal Pkinase_Tyr domain, whereas MUSK_CHICK contains an additional Kringle domain between the Fz and Pkinase_Tyr domains (Figure S4/a). Since all mammalian MUSK orthologs lack kringles, whereas all fish, amphibian and bird orthologs of human MUSK have a kringle domain this indicates that the ancestral form of MUSK had an internal kringle and it was lost early in the mammalian lineage [46].

Comparison of orthologs of DCBD1 (Discoidin, CUB and LCCL domain-containing protein 1) provides an example for a more recent change in DA (TreeFam tree TF330156). DCBD1_MOUSE differs in domain architecture from DCBD1_HUMAN in that it lacks the C-terminal discoidin (F5_F8_type_C) domain (Figure S4/b). Although this domain is present in DCBD1 orthologs of horse, dog, pig, opossum, chicken and frog, it is missing from transcripts of rat and mouse DCBD1 and missing from rat and mouse genomic sequences, suggesting that it was lost in the murine lineage.

Another example of DA change reflecting gene rearrangement is seen in the evolution of neurotrypsin (TreeFam tree TF329295). The domain architecture of vertebrate orthologs of NETR_HUMAN (a kringle domain, four in tandem SRCR domains and a trypsin domain (Figure S4/c) is conserved in fish, frog, birds and all mammals with the exception of rat and mouse. Neutrotrypsins of mouse and rat have only three SRCR domains (no evidence for a fourth SRCR domain in rat and mouse genomic sequences), suggesting that one SRCR domain was lost in the murine lineage [47].

Most examples of domain gain come from comparison of more distantly related orthologs, primarily comparisons across the vertebrate/invertebrate boundary. For example, the vertebrate orthologs of the amyloid precursor A4_HUMAN (TreeFam tree TF317274) have the same domain architecture but differ from those of A4_CAEEL and A4_DROME in containing an internally inserted Kunitz_BPTI domain (Figure S4/d). Since none of the invertebrate orthologs of amyloid precursor A4 (including those from *Trichoplax adhaerens, Nematostella vectensis, Strongylocentrotus purpuratus, Branchiostoma floridae*) were found to contain a Kunitz_BPTI domain, it seems likely that this internal domain was gained in the vertebrate lineage.

A major category of DA alterations includes expansion and shrinkage of tandem arrays of internally duplicated domains. For example, the DAs of DMBT1_HUMAN and DMBT1_MOUSE (TreeFam tree TF329295) differ only in the number of tandem SRCR and CUB domains.

Analysis of the relative frequency of orthologous pairs of Swiss-Prot sequences that differ in the number of domains by 1, 2, 3… N domains revealed that for all species orthologs differed in DA most frequently in single domains (70% of the total number of cases), pairs that differed in two domains (15% of the total number of cases), three domains (5% of the total number of cases)… N domains were increasingly less frequent. For example, in the case of *Mus musculus-Homo sapiens* comparisons 74% of the 167 cases belong to the category where DAs differ in a single domain (Table S2/A).

Analysis of the relative frequency of orthologous pairs that do not differ in the number of domains but differ in the number of types of domains (e.g., ABC↔AFC, ABCD↔AFGD, *etc.*) has failed to identify any true case of domain replacement indicating that domain-replacement is much rarer than gain/loss of domains. The cases identified as belonging to this category proved to be false positives, primarily as a consequence of ambiguity in assignment of Pfam A domains. As a typical example we mention the case of vertebrate agrins (Figure S1). The DA of chick and human agrin, appear to be identical in as much as they align over their entire length. The number of domains in AGRIN_HUMAN and AGRIN_CHICK is identical yet their DAs are identified by Pfam as different even at e-value <10^−1^ since their equivalent/orthologous follistatin domains are sometimes assigned to different Pfam A domain families (Kazal_1 and Kazal_2) of the same domain clan (Kazal). Such a difference might be automatically assigned to the domain-replacement category whereas the truth is that no DA change distinguishes the DAs of agrins of human and chick.

When we classified DA differences of human Swiss-Prot proteins and their Swiss-Prot orthologs from *Pongo abelii, Mus musculus, Gallus gallus, Xenopus tropicalis, Danio rerio, Drosophila melanogaster* and *Caenorhabditis elegans* (a total of 382 cases) with respect to the position of the distinguishing Pfam A domain(s) it was noted that internal Pfam A domain differences were less frequent (6% of total comparisons) than those at N-terminal (24% of total comparisons) or C-terminal positions (14% of total comparisons). The highest proportion of DA differences (26% of total comparisons) originated from gain/loss of tandem duplicated domains. In 10% of comparisons the DA differences was not assigned to any of the above categories (one of the orthologs did not contain a Pfam A domain), whereas 21% of the comparisons yielded identical DA at one of the cut-off values.

To examine whether the greater frequency of terminal DA alterations reflects a greater probability of fusion-type events than insertion-type events or is due to the preponderance of the one-domain ↔ two-domain transition type (where DA change is by definition terminal) we have analyzed the positional distribution for type 1 transitions (one-domain ↔ two-domain transitions), type 2 transitions (two-domain ↔ three-domain transitions) and for type 3 transitions (N-domain ↔ N + 1-domain transitions, where N > 2) separately (Table 2).

This analysis has shown that in the case of type 2 transitions of Swiss-Prot orthologs (where there is an equal number of *N*-terminal, *C*-terminal and internal positions for DA change) the proportion of DA changes at the three different positions is quite similar: on average 25.9%, 24.3% and 29.4% of the DA changes were of the N-terminal, C-terminal and internal type, respectively (Table 2). For example, in the case of DA comparisons of chick-human orthologs DA changes for type 2 transitions were found to occur at the N-terminal, C-terminal and Internal positions in 37%, 21% and 26% of the total comparisons, respectively. Similarly, in the case of comparison of mouse-human orthologs DA changes for type 2 transitions were found to occur at the N-terminal, C-terminal and Internal positions in 25%, 33% and 24% of comparisons, respectively. These observations suggest that the probability of DA change is similar for terminal and internal positions.

Consistent with this interpretation, in the case of type 3 transitions of Swiss-Prot orthologs (where there are more internal than N-terminal or C-terminal positions for DA change) there was a significant shift in favor of internal DA changes: on average 10.2%, 6.2% and 40.3% of the DA changes were of the N-terminal, C-terminal and internal type, respectively. For example, in the case of chick-human orthologs the values for N-terminal-, C-terminal- and internal DA differences were 3%, 6% and 53% and the corresponding values of mouse-human orthologs were 8%, 6% and 45%, respectively. It may be noted that the proportion of DAs that differ only in the number of tandem copies of a Pfam A domain type is higher in the case of type 3 transitions (on average 33.3%) than in the case of type 2 transitions (on average 20.0%). This difference is explained by the fact that the category of type 3 transitions is enriched in multidomain proteins with a large number of domains, many of which contain tandem arrays of the same the domain type (e.g., DMBT1_HUMAN and DMBT1_MOUSE).

In view of the relatively low number of cases where the DA of a human Swiss-Prot entry was found to differ from of its Swiss-Prot ortholog from *Pongo abelii, Mus musculus, Gallus gallus, Xenopus tropicalis, Danio rerio, Drosophila melanogaster* or *Caenorhabditis elegans* (a total of 382 cases) one should be cautious in drawing conclusions as to the relative frequency of the different types of gene rearrangements. Nevertheless, we wish to point out that we noted no preference of terminal over internal DA changes.

**Table 2:** Positional distribution of DA differences observed when sequences from different databases (Swiss-Prot, TreEMBL, RefSeq, EnsEMBL, GNOMON) were compared with sequences of orthologous human Swiss-Prot proteins.

**Database**	Type of DA difference*			

	**Nterm**	**Cterm**	**Internal**	**Duplication**
**Swiss-Prot**				
Type 1 transition	46.74%	27.96%	9.19%	9.19%
Type 2 transition	25.9%	24.3%	29.4%	20%
Type 3 transition	10.2%	6.2%	40.3%	33.3%

**TrEMBL**				
Type 1 transition	43.9	40.8	0	13
Type 2 transition	35.5	31.7	6.8	23.9
Type 3 transition	39.6	17.4	8.9	33.8

**RefSeq**				
Type 1 transition	39.80	33.80	9.98	9.98
Type 2 transition	30.70	22.90	26.60	18.00
Type 3 transition	20.78	11.69	38.49	28.72

**EnsEMBL**				
Type 1 transition	50.9	35.4	0	11.3
Type 2 transition	41.7	25.1	9.2	22.4
Type 3 transition	26.3	15.7	14.8	42.1

**GNOMON**				
Type 1 transition	41.20	32.72	9.96	9.96
Type 2 transition	29.62	20.02	28.50	20.74
Type 3 transition	21.60	15.08	37.29	25.22

* The numbers in the different categories represent the percent of total assignments

### 2.3. Comparison of the DA of Human Swiss-Prot Protein Sequences with Orthologous Metazoan TrEMBL Protein Sequences

Comparison of the DA of TrEMBL sequences of worm, fly and several vertebrate species with those of orthologous/equivalent human Swiss-Prot sequences revealed that the rate of DA deviation was always higher than in the case of Swiss-Prot/Swiss-Prot comparisons (compare Table 1/A and Table 1/B). For example, *Homo-Homo*: 4.8% *vs.* 0.00%; *Homo-Mus*: 4.1% *vs.* 1.1%; *Homo-Gallus*: 4.4% *vs.* 3.0%; *Homo-Xenopus*: 4.7% *vs.* 0.9%; *Homo-Danio*: 5.0% *vs.* 2.1%; *Homo-Drosophila*: 9.4% *vs.* 4.8%; *Homo-Caenorhabditis*: 12.3 *vs.* 5.9% (the first values refer to TrEMBL/Swiss-Prot comparisons, the second values refer to Swiss-Prot/Swiss-Prot comparisons).

The explanation for this difference between Swiss-Prot/Swiss-Prot and TrEMBL/Swiss-Prot comparisons is that the TrEMBL database is significantly contaminated with incomplete (N-terminally or C-terminally truncated) or chimeric protein sequences [3] and this contamination increases the rate of DA differences since the DA of fragments or chimeras differs from the DA of their complete Swiss-Prot orthologs.

Analysis of the relative frequency of orthologous pairs of Swiss-Prot/TrEMBL sequences that differ in the number of domains by 1, 2, 3… N domains revealed that—similarly to the observations on Swiss-Prot/Swiss-Prot comparisons—the pairs differed most frequently in single domains (60% of the 3214 cases analyzed), pairs that differed in two domains, three domains … N domains were increasingly less frequent (Table S2/B). However, in the case of TrEMBL there is a detectable shift in favor of DA differences where the pairs differ in more than one domain: whereas in the case of Swiss-Prot/Swiss-Prot comparisons 30% of the orthologous pairs differed in more than one domain, in the case of Swiss-Prot/TrEMBL pairs this value is 40%. For example, in the case of *Mus musculus-Homo sapiens* comparisons 57.94% of the cases belong to the category where DAs differ in a single domain, whereas this value is 74% in the case of Swiss-Prot/Swiss-Prot comparison. This shift is also explained by the fact that incomplete and chimeric sequences significantly contaminate TrEMBL and these are more likely to differ in multiple domains.

In harmony with this interpretation, comparison of the positional distribution of DA differences in TrEMBL/Swiss-Prot comparisons with those observed in Swiss-Prot/Swiss-Prot comparisons revealed that there is a significant shift in favor of terminal over internal differences. Whereas in the case of type 2 transitions of Swiss-Prot/Swiss-Prot comparisons the proportion of N-terminal (26%), C-terminal (24%) and internal (29%) DA changes were comparable (see Table 2) in the case of TrEMBL/Swiss-Prot comparisons the corresponding values were 36%, 32% and 7%, respectively. The increased proportion of terminal DA changes in TrEMBL/Swiss-Prot comparisons was also obvious in the case of type 3 transitions (Table 2). Whereas in the case of type 3 transitions of Swiss-Prot/Swiss-Prot comparisons the proportion of internal (40%) DA changes exceeded those of the N-terminal (10%) and C-terminal (6%) changes, in the case of TrEMBL/Swiss-Prot comparisons the N-terminal (40%) and C-terminal (17%) DA changes still exceeded the proportion of internal changes (9%).

An inspection of the data shown in Table 2 indicates that in the case of type 1 and type 2 transitions errors affect the N-terminal and C-terminal parts of TrEMBL sequences with roughly similar probability. This observation suggests that although different types of errors contribute to N-terminal and C-terminal DA deviation of TrEMBL sequences (see section 2.1.) their contribution is roughly similar.

In the case of multidomain proteins with a larger number of constituent domains (represented in type 3 transitions, Table 2), however, the ratio of N-terminal *vs.* C-terminal DA change shows a strong preference for DA difference at the N-terminal end, suggesting that larger cDNAs (encoding larger multidomain proteins) are more likely to be incomplete at their 5′ end.

### 2.4. Comparison of the DA of Human Swiss-Prot Protein Sequences with Orthologous Metazoan RefSeq, EnsEMBL and NCBI Protein Sequences

#### 2.4.1. Comparison of the DA of Human Swiss-Prot Protein Sequences with Orthologous Metazoan RefSeq Protein Sequences

Comparison of the DA of human, mouse, chicken, frog, zebrafish, worm and fly protein sequences of the RefSeq database with the corresponding/orthologous human Swiss-Prot entries revealed that, similarly to the case of Swiss-Prot/Swiss-Prot comparisons the percentage of orthologs with different DA increased with the evolutionary distance of the species compared (Table 1/C). It should be noted, however, that in the case of RefSeq/Swiss-Prot comparisons the proportion of domain architecture differences was consistently higher than in the case of Swiss-Prot/Swiss-Prot comparisons (compare Table 1/A and Table 1/C). For example *Homo-Homo*: 0.4% *vs.* 0.00%; *Homo-Mus*: 1.5% *vs.* 1.1%; *Homo-Gallus*: 3.8% *vs.* 3.0%; *Homo-Xenopus*: 3.1% *vs.* 0.9%; *Homo-Danio*: 4.7% *vs.* 2.1%; *Homo-Drosophila*: 10.0% *vs.* 4.8%; *Homo-Caenorhabditis*: 10.9 *vs.* 5.9% (the first values refer to RefSeq/Swiss-Prot comparisons, the second values refer to Swiss-Prot/Swiss-Prot comparisons).

Analysis of the relative frequency of orthologous pairs of Swiss-Prot/Refseq sequence pairs that differ in the number of domains by 1, 2, 3… N domains revealed that pairs differed most frequently in single domains (67% of the 2625 cases analyzed), pairs that differed in two domains (17% of the cases), three domains (6% of the cases)… N domains were increasingly less frequent. Note that these values are similar to those observed in the case of Swiss-Prot/Swiss-Prot comparisons. For example in the case of *Mus musculus-Homo sapiens* comparisons 74% of the cases belong to the category where DAs differ in single domains in both the Swiss-Prot/Swiss-Prot comparisons and Swiss-Prot/RefSeq comparisons (compare Tables S2/A and S2/C).

When we analyzed the positional distribution of DA differences and compared them with those observed in the case of Swiss-Prot/Swiss-Prot comparisons, we noted differences only in the case of type 3 transitions (Table 2). Here the proportion of N-terminal and C-terminal change (20.78% and 11.69%) was higher in the case of RefSeq/Swiss-Prot comparisons than in the case of Swiss-Prot/Swiss-Prot comparisons (10.2% and 6.2%). This shift in favor of terminal DA changes is in harmony with the interpretation that the RefSeq dataset contains some incomplete or mispredicted sequences and these are most likely to differ from the DA of their complete orthologs at the N-terminal or C-terminal ends.

#### 2.4.2. Comparison of the DA of Human Swiss-Prot Protein Sequences with Orthologous Metazoan Gnomon Predicted Protein Sequences

As discussed in section 2.4.1, a major difference between Swiss-Prot and RefSeq databases is that the latter contains a relatively high proportion of hypothetical predicted sequences whereas the majority of Swiss-Prot entries are experimentally verified sequences. It seemed therefore plausible to assume that the higher rate of DA difference observed in Swiss-Prot/RefSeq comparisons *versus* Swiss-Prot/Swiss-Prot comparisons may be due to mispredicted sequences contaminating the RefSeq database.

As a further test of the validity of this explanation we have analyzed a dataset that contained only predicted sequences: the dataset of NCBI's GNOMON-predicted sequences. In harmony with our expectation the rate of DA deviation was found to be higher in comparison of human Swiss-Protein entries with orthologous GNOMON predicted sequences than in comparison of human Swiss-Protein entries with orthologous Swiss-Prot sequences or in comparison of human Swiss-Protein entries with orthologous RefSeq sequences (compare Tables 1/A, 1/C and 1/D).

This difference between Swiss-Prot, Refseq *versus* NCBI's GNOMON-predicted sequences is most obvious when we compare the rate of DA deviation of human entries identified as equivalents of human Swiss-Prot entries: in the case of Refseq database 0.4% of the human Refseq entries differ in DA from a corresponding Swiss-Prot entry whereas it is 7.90% in the case of NCBI's GNOMON-predicted sequences. This tendency is also obvious in the case of *Danio rerio* sequences where the proportion of DA differences is markedly different for Swiss-Prot entries (2.1%), Refseq entries (4.7%) or for NCBI's GNOMON-predicted entries (9.9%).

Analysis of the relative frequency of orthologous pairs of Swiss-Prot/GNOMON sequences that differ in the number of domains by 1, 2, 3… N domains revealed that pairs differed most frequently in single domains (62% of the 2338 cases analyzed), pairs that differed in two domains (18% of the cases), three domains (7% of the cases)… N domains were increasingly less frequent (table S2/D). When we compare these values with the corresponding values for Swiss-Prot/Swiss-Prot comparisons we note a shift in favor of DA changes involving more than one domain. For example in the case of *Mus musculus-Homo sapiens* comparisons only 56% of the cases belong to the category where DAs differ in single domains in Swiss-Prot/GNOMON comparisons whereas in the case of Swiss-Prot-Swiss-Prot and Swiss-Prot/RefSeq comparisons this value is 74% (compare tables S2/A, S2/C and S2/D). This observation is in harmony with the presence of incomplete and mispredicted sequences in this database.

Analysis of the positional distribution of DA differences observed in the case of Swiss-Prot/GNOMON comparisons (Table 2) revealed that it is quite similar to that observed in the case of Swiss-Prot/Swiss-Prot comparison except that in the case of type 3 transitions the proportion of terminal DA changes was higher in the case of Swiss-Prot/GNOMON comparisons than in the case of Swiss-Prot/Swiss-Prot comparisons (N-terminal change: 21.60% *versus* 10.2%; C-terminal change 15.08% *versus* 6.2%). This shift in favor of terminal DA changes probably reflects the presence of mispredicted sequences that are most likely to differ in DA from their complete orthologs at the N-terminal or C-terminal ends.

Similarly to Swiss-Prot/Swiss-Prot and Swiss-Prot/RefSeq comparisons, in the case of Swiss-Prot/GNOMON comparisons N-Terminal DA deviation always exceeds that observed at the C-terminal end. As shown in Table 2 in the case of type 1, type 2 and type 3 transitions of Swiss-Prot/GNOMON comparisons the values for N-terminal and C-terminal DA changes were 41% *vs.* 33%, 30% *vs.* 20%, and 22% *vs.* 15%, respectively. The most plausible explanation for the dominance of DA change at the N-terminal end is that it reflects the fact that N-terminal exons are predicted with lower accuracy than C-terminal exons [6].

#### 2.4.3. Comparison of the DA of Human Swiss-Prot Proteins with Orthologous Metazoan EnsEMBL Sequences

The data obtained by comparison of the DA of human, mouse, chicken, frog, zebrafish, worm and fly protein sequences of the EnsEMBL database with the corresponding/orthologous human Swiss-Prot entries were similar to those obtained in RefSeq/Swiss-Prot and GNOMON/Swiss-Prot comparisons in as much as the percentage of orthologs with different DA increased with the evolutionary distance of the species compared and that the proportion of domain architecture differences was always higher than in the case of Swiss-Prot/Swiss-Prot comparisons (Table 1/E). For example, *Homo-Homo*: 1.09% *vs.* 0.00%; *Homo-Mus*: 1.57% *vs.* 1.1%; *Homo-Gallus*: 3.9% *vs.* 3.0%; *Homo-Xenopus*: 5.76% *vs.* 0.9%; *Homo-Danio*: 3.27% *vs.* 2.1%; *Homo-Drosophila*: 9.01% *vs.* 4.8%; *Homo-Caenorhabditis*: 10.78 *vs.* 5.9%).

Analysis of the relative frequency of orthologous pairs of Swiss-Prot sequences that differ in the number of domains by 1, 2, 3… N domains revealed that pairs differed most frequently in single domains (65% of the 2964 cases analyzed), pairs that differed in two domains (18% of the cases), three domains (7% of the cases)… N domains were increasingly less frequent (Table S2/E). Comparison of these data, with the corresponding values for Swiss-Prot/Swiss-Prot comparisons, indicates that the presence of mispredicted sequences results in a slight shift in favor of DA changes involving multiple domains. For example in the case of *Mus musculus-Homo sapiens* comparisons 66% of the cases belong to the category where DAs differ in single domains in Swiss-Prot/EnsEMBL comparisons, whereas in the case of Swiss-Prot-Swiss-Prot and Swiss-Prot/RefSeq comparisons, this value is 74% (see Table S2).

When we analyzed the positional distribution of DA differences and compared them with those observed in the case of Swiss-Prot/Swiss-Prot comparisons, we noted that in the case of type 2 transitions the proportion of internal change was lower and that of N-terminal changes was higher in the case of EnsEMBL/Swiss-Prot comparisons (Table 2): N-terminal changes: 41.7% *vs.* 25.9%, internal changes: 9.2% *vs.* 29.4% (the first values refer to EnsEMBL/Swiss-Prot comparisons, the second values refer to Swiss-Prot/Swiss-Prot comparisons). The same tendency was also observed in the case of type 3 transitions: in the case of EnsEMBL/Swiss-Prot comparisons the proportion of internal change was lower, those of terminal changes were higher than in the case Swiss-Prot/Swiss-Prot comparisons: N-terminal changes: 26.3% *vs.* 10.2%; C-terminal changes: 15.7%% *vs.* 6.2%; internal changes: 14.8%% *vs.* 40.3% (the first values refer to EnsEMBL/Swiss-Prot comparisons, the second values refer to Swiss-Prot/Swiss-Prot comparisons). Significantly, in the case of Swiss-Prot/EnsEMBL comparisons the proportions of N-terminal and C-terminal DA deviations exceed that observed at internal positions even in the case of type 3 transitions. This is in sharp contrast with the other databases, except the TrEMBL database (Table 2). It seems likely that this similarity of the data obtained on TrEMBL and EnsEMBL databases reflects the fact that EnsEMBL contains sequences predicted by Wise2 that relies on experimental data that are also represented in the TrEMBL database.

### 2.5. Influence of Sequence Errors on Genome-Scale Comparison of Domain Architectures of Proteins

As discussed above, the presence of mispredicted sequences amongst RefSeq, EnsEMBL and GNOMON-predicted sequences increases the apparent rate of DA differences when these sequences are compared with orthologous high quality human Swiss-Prot sequences. In genome-scale studies, however, comparisons usually involve predicted proteomes represented in the RefSeq database, thus it may be expected that the influence of mispredicted sequences on DA differences is even more severe when we compare human RefSeq sequences with orthologous Refseq sequences.

In harmony with this expectation, in the case of all species analyzed the rate of DA deviation is higher in the case of RefSeq/RefSeq comparison than in the case of RefSeq/Swiss-Prot comparisons and much higher than in the cases of Swiss-Prot/Swiss-Prot comparisons (compare Tables 1/A, 1/C and 1/F). For example, in comparison of *Homo sapiens* RefSeq sequences with *Danio rerio* RefSeq sequences the proportion of DA differences is 8.16%, whereas, in the case of Swiss-Prot/RefSeq comparisons, it is 4.7%, and in the case of Swiss-Prot/Swiss-Prot comparisons, the rate of DA difference is only 2.1%.

The apparent rate of DA difference is more exaggerated when human RefSeq sequences are compared with orthologous GNOMON-predicted sequences (compare Table 1/A, Table 1/C, Table 1/F and Table 1/G). For example, in comparison of *Homo sapiens* RefSeq sequences with orthologous *Danio rerio* GNOMON predicted sequences 14.22% of the orthologous sequences were found to show a DA difference.

It must be pointed out that in the case of comparison of human Refseq sequences with orthologous GNOMON predicted sequences the DA difference significantly exceeds those observed in the case of Swiss-Prot/Swiss-Prot comparisons. For example, *Homo-Mus*: 9.3% *vs.* 1.1%; *Homo-Gallus:* 8.0% *vs.* 3.0%; *Homo-Danio*: 14.2% *vs.* 2.1%; *Homo-Drosophila*: 14.3% *vs.* 4.8%; *Homo-Caenorhabditis*: 16.6% *vs.* 5.9% (the first values refer to Refseq/GNOMON comparisons, the second values refer to Swiss-Prot/Swiss-Prot comparisons). In other words, the DA differences due to contamination of databases with mispredicted sequences exceed those that result from genomic rearrangements.

The increase in DA difference due to sequence errors is most spectacular in the case of human/vertebrate comparisons but less striking in the case of human/invertebrate comparisons. This difference is probably explained by the fact that the problems of gene prediction are less severe in the case of invertebrates (such as worms and fly) that have less intron-rich genomes than vertebrates.

These studies indicate that when predicted proteomes are compared the rate of DA difference resulting from misprediction may be higher than that arising from gene rearrangements. As a corollary, the influence of evolutionary distance on DA difference observed in the case of Swiss-Swiss-Prot, Swiss-Prot/RefSeq comparisons is barely detectable in the case of RefSeq/GNOMON comparisons (compare Table 1/A, Table 1/C and Table 1/G).

### 2.6. Identification and Correction of Erroneous Sequences that Differ in DA from Their Human Orthologs

To test our explanation that mispredicted sequences account for the increased rate of DA deviation observed in the case of Swiss-Prot/RefSeq, Swiss-Prot/EnsEMBL, Swiss-Prot/NCBI, RefSeq/RefSeq and RefSeq/GNOMON comparisons we focused on proteins where orthologous pairs differed in DA only in the case of these comparisons but not in the case of Swiss-Prot/Swiss-Prot comparisons (suppressing the contribution of true positives reflecting alternative splicing and genomic rearrangement).

EnsEMBL, RefSeq and NCBI/GNOMON sequences thus selected were subjected to in-depth analysis (as described in the Experimental Section). These analyses have confirmed that in the case of comparisons of human Swiss-Prot entries with orthologous EnsEMBL, RefSeq or GNOMON-predicted sequences the DA differences are primarily due to sequence errors (mispredicted, incomplete or abnormal sequences). In the case of some genes and genomes (chicken, frog, zebrafish genomes) misprediction was ‘forced’ in the sense that it was a consequence of the relatively poor quality of the genome sequence (presence of unsequenced regions) rather than the poor performance of gene prediction methods.

As an example we may quote the case of XP_426568, the chicken ortholog of FZD8_HUMAN. FZD8_HUMAN and its orthologs all contain an Fz and a Frizzled domain (as well as a signal peptide). In contrast with this, the chicken ortholog predicted by GNOMON (XP_426568) lacks the N-terminal Fz domain (Figure S5). The fact that the Frizzled domain of this protein is N-terminally truncated (violation of MisPred rule 4; [3]) cautioned that the protein is mispredicted. Our FixPred protocol (see Experimental Section) failed to correct this misprediction since no EST supporting the correct N-terminal region was found and the genomic region containing this gene on chromosome 7 contained a large unsequenced region just upstream of the point where the Frizzled domain was truncated. Note that the forced misprediction resulted in a DA change that appears as a DA change of the N-terminal type.

In the majority of cases, however, prediction errors could be corrected by the FixPred protocol. This point may be illustrated by examples that come from analysis of orthologs of the type I transmembrane protein KREM1_HUMAN and KREM2_HUMAN (TreeFam tree TF331319). KREM1_HUMAN, KREM1_MOUSE, KREM1_RAT, KREM1_XENLA and KREM2_HUMAN, KREM2_MOUSE have identical DA: they all contain a Kringle, a WSC and a CUB domain (as well as a signal peptide and a transmembrane segment). In contrast with this, the Refseq ortholog of kremen 1 from *Xenopus tropicalis* (NP_001116927) lacks an N-terminal kringle domain. The fact that the protein also lacks a signal peptide cautions that it is N-treminally truncated (violation of MisPred rule 1; [3]). Analysis of the genome of *Xenopus tropicalis* and EST databases permitted the correction of the prediction with the help of the FixPred protocol (Figure S6). Note that the apparent DA change due to this sequence error (incomplete sequence) appears as a DA change of the N-terminal type.

The DA of the Refseq ortholog of kremen 2 from *Xenopus tropicalis* (NP_001072931) differs from those of its orthologs and paralogs in that it is C-terminally truncated therefore it lacks the C-terminal CUB domain (as well as the transmembrane region). The C-terminal part of the WSC domain is also missing from this hypothetical protein (violation of MidsPred rule 4; [3]) cautioning that the C-terminal part may be incorrect (note that despite the truncation the WSC domain is detected by Pfam). In-depth analysis of the genomic region has revealed that the transcript (NM_001079463) encoding this protein arose as a result of an aberrant splicing of a phase 0 intron within the region encoding the WSC domain. Instead of the normal 3′ splice site of the intron with the correct phase (phase 0) the splicing occurred at a downstream low probability splice site of an incorrect phase (phase 1), resulting in a frame-shift and C-terminal truncation of the WSC domain and ‘deletion’ of the downstream CUB domain. Note that the apparent DA change due to this error (abnormal transcript) appears as a DA change of the C-terminal type. Analysis of the genome of *Xenopus tropicalis* and EST databases permitted the correction of this incomplete sequence with the help of the FixPred protocol (Figure S7).

In some cases, prediction errors could be corrected by the FixPred protocol simply by tiling of ESTs. For example, the DA of the GNOMON-predicted sequence of the chicken protein XP_416936 differs from that of its ortholog GAS6_HUMAN: whereas GAS6 proteins contain a signal peptide, a Gla, three EGF_CA, a Laminin_G_1 and a Laminin_G_2 domain, XP_416936 lacks the N-terminal signal peptide and Gal domain. The fact that the protein lacks a signal peptide cautions that it is N-terminally truncated (violation of MisPred rule 1; [3]) and that the DA deviation reflects a sequence error rather than a true change in genomic structure. A search of EST databases has identified ESTs CD217792, BM439645 and BU115578 that permitted the correction of the sequence to include the missing signal peptide and Gla domains (Figure S8). Note that the apparent DA change due to this error in gene prediction appears as a DA change of the N-terminal type.

**Figure 1:**
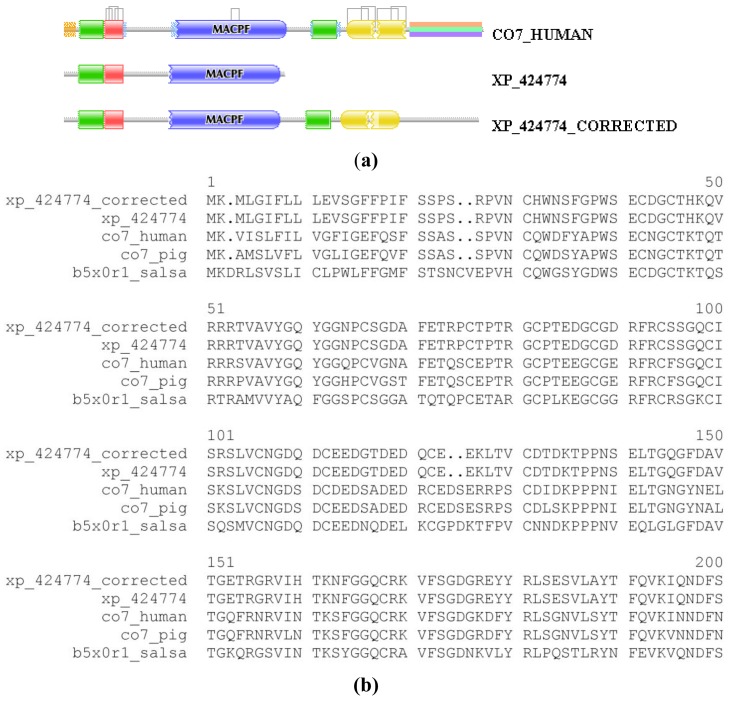

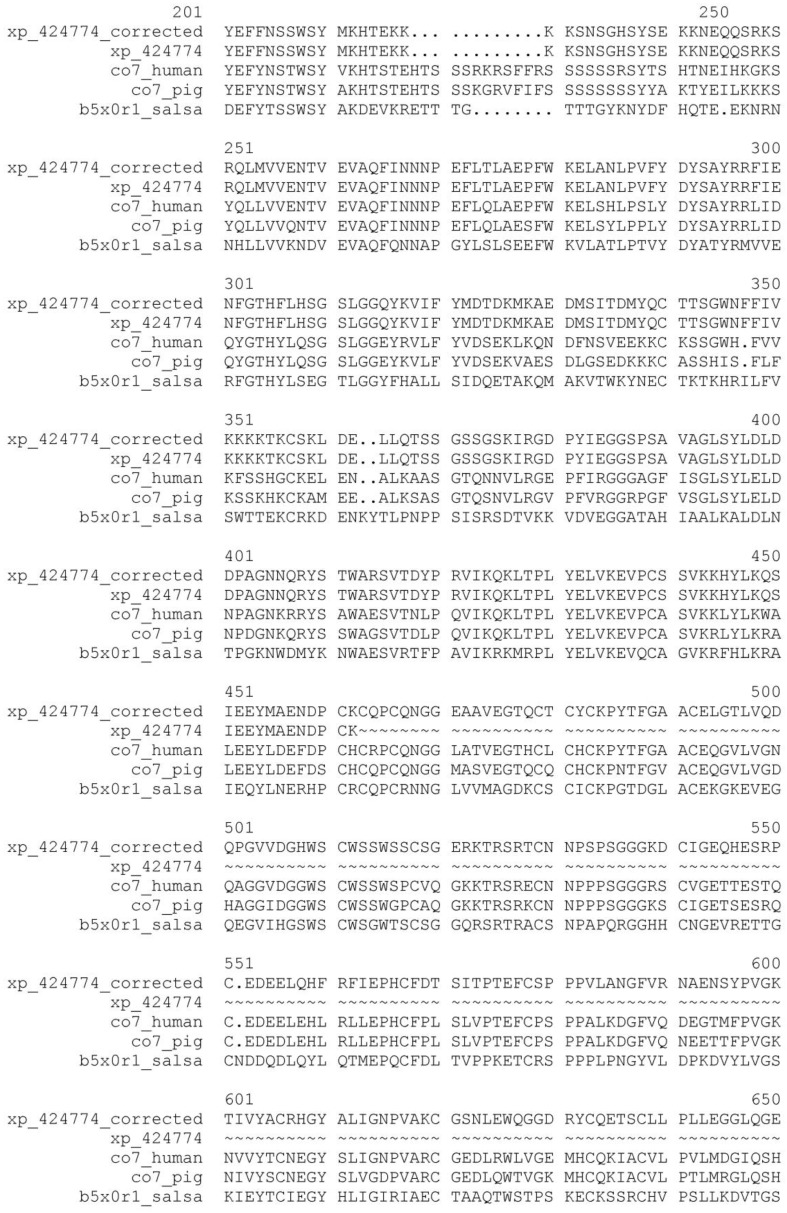

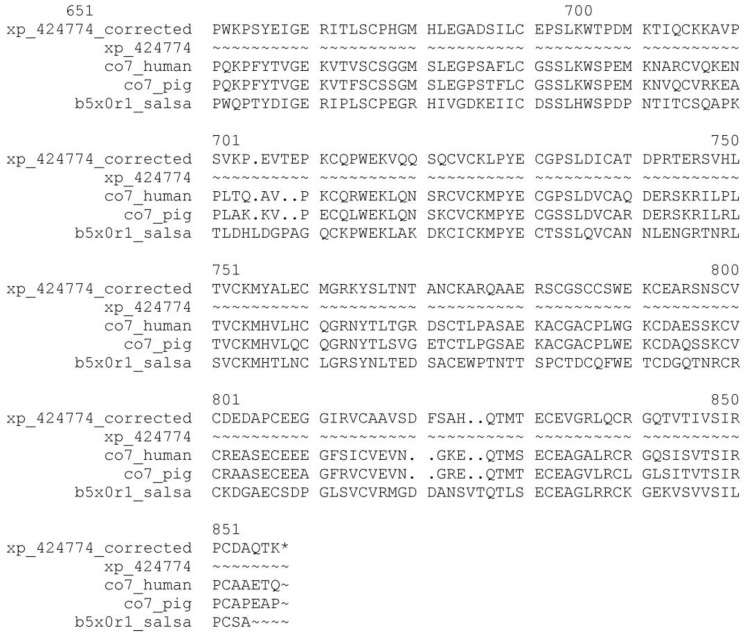
Correction of the sequence of complement component C7 of *Gallus gallus* with the FixPred protocol. The DA of GNOMON-predicted sequence of complement component C7 from Gallus gallus (XP_424774) was found to differ from those of its mammalian and fish orthologs (CO7_HUMAN, CO7_PIG, B5X0R1_SALSA): whereas the latter contain TSP_1, Ldl_recept_a, MACPF, TSP_1, Sushi and Sushi domains the ortholog of Gallus gallus lacks the domains downstream of the MACPF domain. The sequence “XP_424774_CORRECTED” was predicted by the use of alternative gene models and is supported by ESTs. The sequence predicted by FixPred was experimentally verified by cloning the full-length cDNA; the cDNA sequence was deposited in GenBank (accession cDNA: HQ878377; accession protein: ADY17228). (**a**) Comparison of the DAs of XP_424774 and XP_424774_CORRECTED with that of CO7_HUMAN; (**b**) Alignment of the sequences of XP_424774 and XP_424774_CORRECTED with those P_416936, XP_416936_CORRECTED with those of CO7_HUMAN, CO7_PIG and B5X0R1_SALSA.

We illustrate the reliability of the combined use of the MisPred and FixPred protocols with the example of the chicken ortholog of human complement C7. As shown in Figure 1, the sequence of Complement C7 of *Gallus gallus* predicted by NCBI's GNOMON protocol (XP_424774) is C-terminally truncated when compared with its mammalian and fish orthologs: whereas the DA of the latter contain TSP_1/Ldl_recept_a/MACPF/TSP_1/Sushi/Sushi domains, the chicken protein lacks the three domains downstream of the MACPF domain. Analysis of the genome of *Gallus gallus* and EST databases permitted the correction of this incomplete sequence with the help of the FixPred protocol and the prediction was verified by cloning the full-length cDNA of the protein (Figure 1). Note that the apparent DA change due to this error in gene prediction appears as a DA change of the C-terminal type involving multiple domains.

**Figure 2:**
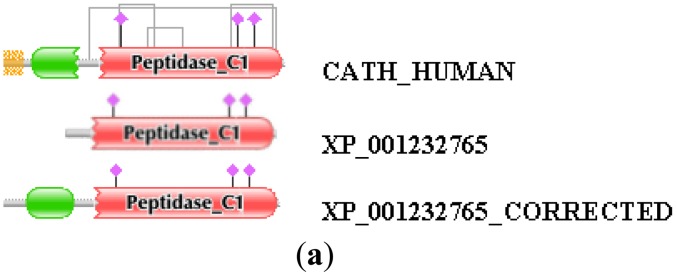

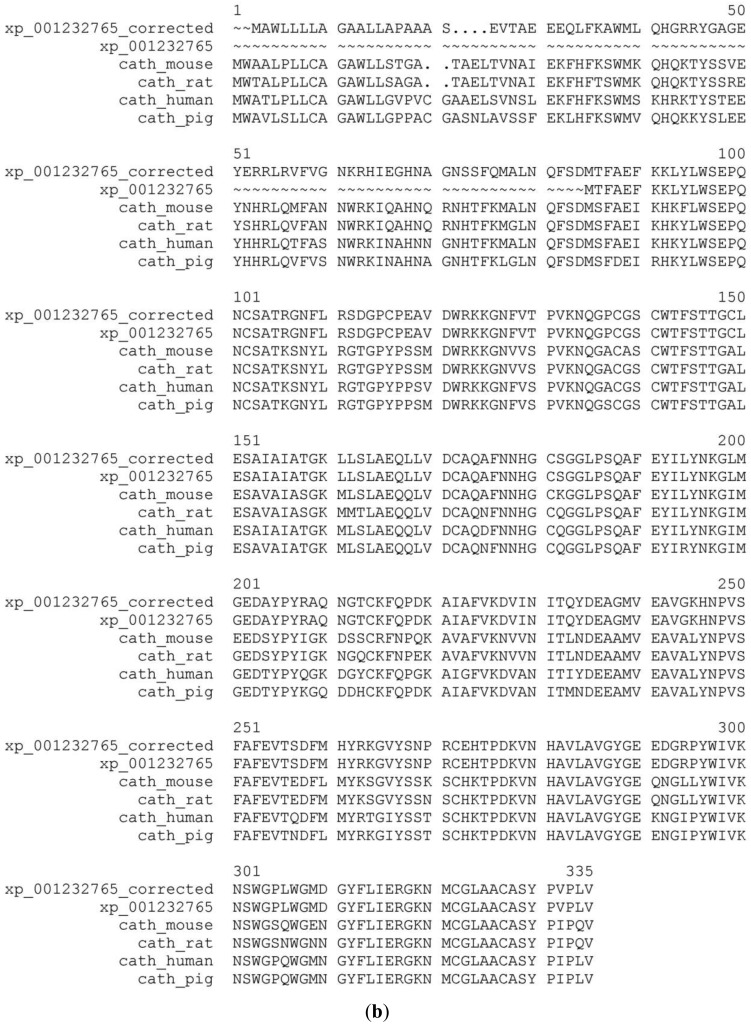
Correction of the sequence of cathepsin H of *Gallus gallus* with the FixPred protocol. The DA of GNOMON-predicted sequence of cathepsin H from *Gallus gallus* (xp_001232765) was found to differ from those of its mammalian orthologs (CATH_HUMAN, CATH_MOUSE, CATH_PIG, CATH_RAT: whereas the DA of the latter contains an Inhibitor_I29 and a Peptidase_C1 domain, the chicken protein lacks the Inhibitor_I29 domain. The sequence “xp_001232765_corrected” was predicted by the use of ESTs bm427347, bi066433, am064052, bu425005 and bi064908. The sequence predicted by FixPred was experimentally verified by cloning the full-length cDNA; the cDNA sequence was deposited in GenBank (accession cDNA: JF514547; accession protein: AEC13302). (**a**) Comparison of the DAs of XP_001232765 and XP_001232765_CORRECTED with that of CATH_HUMAN; (**b**) Alignment of the sequences of XP_001232765 and XP_001232765 _CORRECTED with those of CATH_HUMAN, CATH_MOUSE, CATH_PIG, CATH_RAT.

Similarly, the reliability of the combined use of the MisPred and FixPred protocols may be illustrated with the example of the chicken ortholog of human cathepsin H. As shown in Figure 2, the sequence of cathepsin H of *Gallus gallus* predicted by NCBI's GNOMON protocol (XP_001232765) is N-terminally truncated when compared with its mammalian orthologs: whereas the DA of the latter contain an Inhibitor_I29 and a Peptidase_C1 domain, the chicken protein lacks the Inhibitor_I29 domain. The fact that unlike the mammalian orthologs the chicken protein XP_001232765 lacks a signal peptide is another indication that the sequence is mispredicted (violation of MisPred rule 1; [3]). Analysis of the genome of *Gallus gallus* did not permit the correction of this incomplete sequence since the appropriate genomic region contained a large unsequenced region (a case of forced misprediction). Correction was made possible by the use of EST sequences and the prediction was verified by cloning the full-length cDNA of the protein (Figure 2). Note that the apparent DA change due to this error in gene prediction appears as DA change of the N-terminal type.

## 3. Experimental Section

### 3.1. Databases

UniProtKB Swiss-Prot and UniProtKB TrEMBL entries [48] were downloaded from [49]. Protein sequences were retrieved from the RefSeq database [50,51], the EnsEMBL database [52,53] and NCBI's databases [54,55]. In order to analyze only the sequences predicted by GNOMON [56] an in-house program was used to extract only GNOMON-predicted FASTA sequences with ‘XP_’ identifiers.

### 3.2. Datasets of Orthologous Protein Sequences of Metazoa

The datasets of orthologous Swiss-Prot, TrEMBL, RefSeq, EnsEMBL and NCBI's GNOMON predicted sequences of Metazoan species were created as described in Supplementary Materials.

### 3.3. Comparison of the Domain Architecture of Proteins

#### 3.3.1. Creation of Datasets to Monitor the Specificity and Sensitivity of Protocols Used for Detection of Differences in Domain Architecture

The specificity and sensitivity of alternative approaches used for the detection of architecture differences were monitored on two datasets: one containing orthologous Swiss-Prot entries known to have identical domain architecture and an artificial dataset of orthologous Swiss-Prot entries known to have different domain architecture.

First, we have randomly selected 500 pairs of orthologous human, pongo, rat, mouse, chick, frog, zebrafish, worm and fly Swiss-Prot sequences from the list of orthologs and retained only pairs that align over their entire length (they do not differ in length by more than 30 residues): the pairs thus selected had identical domain architecture as evidenced by Swiss-Prot annotation. This ‘dataset of pairs with identical DA’ was used to assess the false positive rate (α) and specificity (1−α) of detection of domain architecture differences from the equation α = FP/(FP + TN). In these calculations FP (False Positive) equals the number of entries that were identified with a given method as differing in domain architecture (although they have the same domain architecture), whereas TN (True Negative) equals the number of entries that were not identified by the method as differing in domain architecture (and they do not differ in architecture).

Second, to mimic changes in domain architecture of homologous proteins we have generated a dataset of 500 sequences by deleting (or inserting) randomly selected Pfam A domains from or into various (internal or terminal) positions of one member of the pair of orthologous sequences. This dataset was used to assess the false negative rate (β) and sensitivity (1-β), calculated from the equation β = FN/(TP + FN), based on comparison of the proteins of altered DA with their original orthologs. In these calculations FN (False Negative) equals the number of entries that were not identified with the given method as differing in domain architecture from their orthologs (although they do differ), whereas TP (True Positive) equals the number of altered entries that were correctly identified by the method as differing in architecture from their original orthologs.

Sequences with artificially altered DA were also used to test the influence of domain architecture changes on the reliability of the best-hit approach used in this study to establish orthology. In these tests the “parent sequences” were replaced by the “derived sequences” in the original collection of Swiss-Prot entries and the best-hit procedure was repeated. Our results confirmed that correct orthology assignment by the triangulation method is insensitive to changes in domain architecture.

#### 3.3.2. Optimization of the Protocol Used for Detection of Differences in Domain Architecture

First, the domain architectures of the collection of orthologous Swiss-Prot entries with identical domain architectures were determined by RPS-BLAST against the Conserved Domain Database [10,57]. In these searches Pfam-derived position-specific scoring matrices were used and Pfam A domain hits with E values of <10^−9^, <10^−8^, <10^−7^, <10^−6^, <10^−5^ were recorded. In the case of overlapping hits the hits with the lowest E value were selected.

In the second step, the DAs of orthologs were compared and the cases identified as differing in architecture (false positives) were subjected to in-depth analyses using additional tools of domain identification, including Pfam [8,9] and SMART [58,59]. These analyses have revealed that a major source of error is that a Pfam A domain identified in ortholog A remained undetected in ortholog B even at the least stringent (E-value <10^−5^) cut-off value used. This type of error occurred primarily in the case of small domains (e.g., EGF-domains) or domains of low conservation where E-values tend to be close to the least stringent cut-off value. For example, although AGRIN_CHICK and AGRIN_HUMAN align over their entire length, at e-values of <10^−2^, all four EGF domains are detected in the AGRIN_CHICK, whereas only three of these domains are detected in AGRIN_HUMAN (see Figure S1).

To decrease the number of false positives due to this type of error, we have tested protocols in which an additional step was included: domain architectures of positives from the CDD step were determined using the programs of the HMMER 2.3.2 software package and the Pfam HMM libraries [8] and domain hits with less stringent cut-off values were also recorded. Frequently, the use of less stringent criteria for detection of domains failed to eliminate false positives since it led to the detection of additional domain(s) in ortholog A (or B) that remained undetected in ortholog B (or A) *etc.* To overcome this problem we have tested protocols in which two domain architectures were considered to be identical if they had the same number and sequential order of Pfam A domains with at least one of the E-value score cut-off value ranges of <10^−9^, <10^−8^, <10^−7^, <10^−6^, <10^−5^, <10^−4^, <10^−3^, <10^−2^ even if they differed at any single cut-off value. According to this protocol, if sequence A had the same domain architecture at <10^−7^ as sequence B at <10^−4^, then it was concluded that they have the same domain architecture.

A less significant source of false positives was that equivalent domains of orthologous proteins gave best hits with different families of domain clans (EGF *vs.* EGF_CA, Kazal_1 *vs.* Kazal_2 *etc.*). For example, AGRIN_HUMAN and AGRIN_CHICK appear to have different DA because Pfam assigns their equivalent Kazal domains to different families (Kazal_1 and Kazal_2) of the Kazal clan (see Figure S1.). To decrease the number of false positives due to this type of error, we have also tested protocols in which an additional step was included: domain architectures of positives were recalculated using Pfam A domain clans [8].

Next, the various protocols for detection of DA differences were tested on a dataset of orthologous Swiss-Prot entries with artificially altered domain organization to determine their efficiency to detect these DA differences. Not unexpectedly, protocols that lowered false positive rate (and increased specificity) of the detection of DA differences had an opposite effect on false negative rate (and sensitivity): comparison of the specificity and sensitivity of the various protocols revealed that protocols with higher specificity (lower false positive rate) had lower sensitivity (higher false negative rate) and vice versa.

The protocol with the highest average sensitivity and specificity value of (Sn + Sp) / 2 = (0,865 + 0, 985) / 2 = 0.925 was selected to compare the domain architecture of proteins. This protocol consisted of the following steps:

The protein sequences were searched for the presence of domains using RPS-BLAST against the Conserved Domain Database using Pfam-derived position-specific scoring matrices. Domain hits with an e-value of <10^−5^ were recorded, overlapping hits were eliminated and the DA (linear sequence of domains with e value of <10^−5^) was determined. The DA of orthologous pairs was compared and in the case of DA difference their DA was recalculated using the programs of the HMMER 2.3.2 software package and the Pfam HMM libraries at four different e-value cut-offs: <10^−2^, <10^−3^, <10^−4^ and <10^−5^.

#### 3.3.3. Classification of Differences in Domain Architecture

As mentioned above homologous sequence pairs identified by our protocol as differing in DA at e-value <10^−5^ were also analyzed by comparing their DA at less stringent cut-off values (<10^−2^, <10^−3^, <10^−4^). On the basis of the results of these analyses DA differences were classified with respect to:

##### (a) Number of Pfam A domains distinguishing DAs

The motivation for this classification is to define the frequency distribution of one-, two-, three-domain, *etc.* differences (e.g., A↔AB, A↔ABB, A↔ABBC), its dependence on evolutionary distance of the sequences compared, its dependence on the quality of sequences compared, *etc.* Note that this parameter will not detect DA changes that lead to no change in the number of constituent domains (e.g., domain-replacements, such as ABC↔AFC).

##### (b) Number of Pfam A domain-types distinguishing DAs

Note that in the case of simple gain/loss of domains (e.g., A ↔ AB, A↔ABB, A↔ABBC) this parameter is the same as the first parameter, but DA changes that lead to no change in the number of constituent domains (e.g., domain-replacements, such as ABC↔AFC) will also be detected: ABC↔AFC is calculated to differ in domains B and F. The goal of this analysis is to asses the relative frequency of domain replacement *vs.* domain gain/loss and its dependence on evolutionary distance of the sequences compared, its dependence on the quality of sequences compared *etc*.

##### (c) Positions of Pfam A domains that distinguish the DAs

The motivation for this classification is to get an insight into the relative contribution of different genetic mechanisms to DA changes. The rationale is that different genetic mechanisms have different position-preferences: whereas gene-fusion and fission may results in the addition/deletion of domain(s) only at the N-terminal or C-terminal end, domain-shuffling (e.g., through exon-shuffling) does not have this restriction.

In this analysis we examined the position of the distinguishing Pfam A domain(s) relative to shared domain(s). Based on this analysis the pairs of homologs were assigned to the following categories:

1. One member of the pair has extra Pfam A domain(s)–different from the adjacent Pfam A domain-type(s)–at the N-terminal end of shared domain(s). This type of difference is classified as N-Terminal Domain Difference (e.g., B↔ AB or AB ↔ CAB).
2. One member of the pair has extra Pfam A domain(s)–different from the adjacent Pfam A domain-type(s)–at the C-terminal end of shared domain(s). This type of difference is classified as C-Terminal Domain Difference (e.g., A ↔ AB or AB ↔ ABC).
3. One member of the pair has extra Pfam A domain(s)-different from the adjacent Pfam A domain-types(s)–between shared Pfam A domains. This type of difference is classified as Internal Domain Difference (e.g., AB ↔ ACB).
4. One member of the pair has an extra Pfam A domain–identical in type with an adjacent Pfam A domain. This type of difference is classified as Domain Duplication Difference (e.g., AB ↔ ABB).
5. If one member of the pair did not contain any Pfam A domain the relative position of the Pfam A domain in the other homolog was not assigned to any of the above categories. These pairs were entered into the Positionally Not Assigned category.
6. The two members of the pair had identical DA at a given cut-off value. These data were entered into the Identical Domain Architecture category.

Note that since our protocol of DA comparison uses four different cut-off values, four assignments are made for each ortholog pair. In the most unambiguous cases of DA differences the given pair is assigned four times to the same category but in many cases the pair may be assigned to different categories at different cut-off values. Also note that a given pair may show more than one type of difference therefore the given pair may be assigned to more than one category, therefore the sum-total of the assignments may be greater than 4-times the number of pairs compared. For example, the pair ABCDE ↔ BCD (change at both termini) is assigned to both category 1 and category 2.

It may be pointed out that the classification according to the positions of Pfam A domains that distinguish the DAs may introduce a positional bias even if we assume that the probability of DA changes are similar at all positions of the multidomain protein outside the domain boundaries (note that domain-shuffling rarely inserts domains within domain boundaries). As a corollary, in the case of DA changes of the one-domain ↔ two-domain transition-type (herefater called type 1 transition), DA change by definition can only be classified as terminal (e.g., A ↔ AB or A ↔ BA).

Since mathematical analyses of the distribution of multidomain proteins according to the number of different constituent domains have revealed that their distribution follows a power law, *i.e.*, single-domain proteins are the most abundant, whereas proteins containing larger numbers of domains are increasingly less frequent [2] this fact introduces a strong bias in favor of terminal changes that has nothing to do with the probability of genetic mechanisms responsible for internal *vs.* terminal changes.

In contast with this, in the case of two-domain ↔ three domain transitions (e.g., AB ↔ ABC; AB ↔ ACB; AB ↔ CAB, hereafter referred to as type 2 transitions), the distribution is unlikely to be biased, whereas in the case of three-domain ↔ four-domain transitions (e.g., ABC ↔ ABCD; ABC ↔ ABDC; ABC ↔ ADBC; ABC ↔ DABC) and in the case of multidomain proteins with a large number of constituent domains internal changes may become increasingly favored (more internal than terminal positions).

In order to analyze the contribution of this factor to the positional distribution of DA changes, we have also categorized single domain DA changes whether they belong to the one-domain ↔ two domain transitions (type 1 transitions), the two-domain ↔ three domain transitions (type 2 transitions) and the N-domain ↔ N+1-domain transitions, where N is greater than 2 (type 3 transitions).

### 3.4. Simulation of Gene Prediction Errors

To study the influence of gene prediction errors (failure to find true exon, erroneous inclusion of a false exon, misprediction of an exon, fusion of exons of neighboring genes, identification of different parts of a single gene as distinct genes, *etc.*) on domain architecture of hypothetical proteins we have generated datasets of sequences from human Swiss-Prot entries that mimic these errors as described previously [3]. To test the effect of terminal deletions, a group of datasets was created through deletion of 50, 100, 150, 200, *etc.* residues from their N-terminal end or their C-terminal end. Another group of datasets were obtained by deleting the second, third, fourth, *etc.* 50 or 100 residue-segments of the proteins to study the effect of internal deletions. Terminal extensions or internal insertions were simulated by addition/insertion of 50 or 100 amino acid segments (with random sequences and average amino acid composition) to the N-terminal and C-terminal end or after positions 50, 100, 150, *etc.* of these proteins.

A dataset was also generated by fusing randomly selected proteins to a different set of randomly selected proteins to mimic the effect of gene fusions. To mimic erroneous inclusion of true protein-coding exons we have generated a dataset by artificial (terminal or internal) insertion of 100 residues taken at random from other proteins.

Note that these datasets mimic only gene prediction errors that do not disrupt the reading frame. Obviously, mispredictions that result in reading frame-shift will lead to truncation downstream of the point of such misprediction and are likely to lead to C-terminal DA change.

### 3.5. Correction of Erroneous Sequences

We have used the MisPred/FixPred pipeline for the correction of erroneous sequences. As outlined in a previous paper, the MisPred protocol is useful not only for the identification of sequence errors but it also guides the correction of errors [3].

In the case of DA deviation of closely related orthologous sequences the FixPred protocol first tests whether the DA difference is valid or not. If the ‘suspicious’ sequence gives significant alignment over its entire length and in-depth analysis with Pfam rejects DA deviation it is assigned to the false positive category. Conversely, if the region containing the Pfam A domain responsible for the deviation is missing from the other ortholog it is assigned to the true positive category.

In the case of true positives it is first tested whether there is evidence for the existence of sequence versions of the orthologs (in other experimental sequence databases) that do not differ in DA. If search of various protein and nucleic acid sequence databases provides experimental evidence for the expression of such sequences it is concluded that the DA difference observed results either from a sequence error (incomplete, aberrant sequence) or alternative splicing. (Note that predicted sequences are disregarded in this step.)

If the previous steps fail to find experimental evidence for sequence versions that do not differ in DA from its orthologs it is tested whether there is genomic and/or EST evidence for the presence of the domain that is missing from one of the orthologs.

Accordingly, the genomic region containing the gene for the suspicious sequence is subjected to gene-prediction tools and it is asked whether there are alternative predictions that eliminate the DA deviation. In this step we employ AUGUSTUS [60,61], Wise2 [62,63], GenomeScan [64,65] and Fgenesh+ [66,67].

If such predictions are found, it is concluded that no DA change occurred at the genome level. Conversely, if no viable gene models are found that eliminate DA deviation, it is concluded that a genomic rearrangement may underlie the observed DA change. It must be pointed out however, that failure to eliminate DA deviation by alternative gene prediction does not necessarily mean that a DA change resulted from genomic rearrangement: sometimes the missing domain is hidden in unsequenced genomic regions. As we have emphasized in a previous paper, another major source of gene structure errors is the incorrect assembly of genomic contigs [3]. In such cases, the FixPred protocol may still correct sequence errors by using EST sequences (for some illustrative examples see Results).

The reliability of the FixPred protocol was checked experimentally in a few cases by cloning full-length cDNAs of genes whose mispredicted sequences were corrected by the FixPred protocol. Sequences corrected by the FixPred protocol are deposited in the FixPred database [68], whereas corrected FixPred predictions verified experimentally are also deposited in Genbank.

## 4. Conclusions

We have shown that in the case of DA comparisons involving Uniprot/TrEMBL, RefSeq, EnsEMBL and NCBI's GNOMON predicted orthologous protein sequences of Metazoan species the contribution of erroneous (incomplete, abnormal, mispredicted) sequences to domain architecture differences of orthologous proteins may be greater than those of true gene rearrangements. In other words, if we observe a difference in the DA of orthologous predicted sequences it is more likely to reflect a sequence error than true genomic rearrangements.

A practical consequence of this observation is that the domain architecture comparison protocol described in the present work may serve as a tool for the quality control of gene predictions and may also guide the correction of sequence errors (as illustrated in Figures 1 and 2, as well as in Figures S2, S3, S6, S7 and S8). It should be pointed out that the theoretical basis of this quality control tool is related to but distinct from those included previously in our MisPred protocol [3]. The first version of the MisPred approach used five distinct routines for identifying abnormal, incomplete or mispredicted entries based on the principle that a sequence is likely to be incorrect if some of its features conflict with our current knowledge about protein-coding genes and proteins: (i) conflict between the predicted subcellular localization of proteins and the absence of the corresponding sequence signals (MisPred rule 1); (ii) presence of extracellular and cytoplasmic domains and the absence of transmembrane segments (MisPred rule 2); (iii) co-occurrence of extracellular and nuclear domains (MisPred rule 3); (iv) violation of domain integrity (MisPred rule 4); (v) chimeras encoded by two or more genes located on different chromosomes (MisPred rule 5).

An important implication of our findings is that the presence of erroneous sequences in public databases may have led to some erroneous conclusions about the DA evolution of multidomain proteins. First, confusion of DA changes due to sequence errors with those resulting from genomic rearrangements results in a significant overestimation of the rate of DA change during evolution of multidomain proteins. More importantly, we have shown that erroneous sequences are more likely to differ in DA from the correct sequence at terminal than internal positions, thus the use of sequence databases contaminated by erroneous sequences introduce a significant bias in favor terminal over internal DA changes and may lead to erroneous conclusions about the mechanisms involved in DA evolution of multidomain proteins.

In view of these findings we present a reassessment of the DA evolution of multidomain proteins in an accompanying paper [7].

## Supplementary Materials

1. **Creation of Datasets of Orthologous Protein Sequences of Metazoa** The simplest BLAST-based approach used for the identification of orthologs is the RBH (reciprocal best-hit) method [69–72]. The rationale of this approach is that when sequences from two complete proteomes are compared orthologs give reciprocal best hits (*i.e.*, the first sequence finds the second sequence as its best hit in the second species, and vice versa). The most important limitation of the reciprocal best match approach is that it may lead to erroneous conclusions if the proteomes compared are incomplete (or if gene loss has occurred since the speciation event linking the two genomes): instead of the (missing) orthologs, paralogs may be each other's best match. Another limitation of the RBH approach is that if, following divergence of species, the orthologous genes were duplicated in one or both species (1:2, 2:3 orthology *etc.*), the approach will identify just one member of the co-orthology groups as the reciprocal best match. It must be pointed out that in this case the conclusion of orthology from reciprocal best-match analysis will not be erroneous but fails to identify all co-orthologs. 2. **Dataset of Orthologous Swiss-Prot Entries** In the case of the Swiss-Prot database only the human proteome can be considered essentially complete: the current estimate of the gene content of the human genome is ∼ 20,500 protein-coding genes [73] nearly identical with the number of human Swiss-Prot entries in UniProtKB Swiss-Prot (20,331). In the case of the other species, however, only a fraction of the proteomes is represented in Swiss-Prot, therefore the RBH approach is likely to confuse orthology and paralogy. To overcome this problem we used a triangulation method in which human Swiss-Prot entries (present in the essentially complete human Swiss-Prot dataset) served as external reference. The rationale of this approach is that if sequence A of species X and sequence B of species Y (present in incomplete proteomes of these species) give best match with the same human Swiss-Prot sequence (sequence C) in the complete human dataset, then A, B and C belong to the same orthology group. To permit statistically significant analyses we have included only Metazoan species that have at least 1000 Swiss-Prot entries. The species analyzed were: *Homo sapiens* (20,331 entries), *Pongo abelii* (2,184 entries), *Mus musculus* (16,072 entries), *Rattus norvegicus* (7,285 entries), *Gallus gallus* (2,089 entries), *Xenopus tropicalis* (1,378 entries), *Danio rerio* (2,374 entries); *Caenorhabditis elegans* (3,212 entries) and *Drosophila melanogaster* (2,883 entries). Accordingly, to establish orthology relationship between Swiss-Prot entries of *Homo sapiens, Pongo abelii, Mus musculus, Rattus norvegicus, Gallus gallus, Xenopus tropicalis, Danio rerio, Caenorhabditis elegans* and *Drosophila melanogaster* we blasted Swiss-Prot entries against human Swiss-Prot entries and assumed that entries giving the best match (E value cut-off <10^−5^) with the same human Swiss-Prot entry belong to the same orthology cluster. The reliability of this approach is supported by the fact that orthologs identified in this way are in harmony with Swiss-Prot annotation. Analyses of the orthologous clusters identified by the triangulation method revealed that in the case of the Tetrapod species compared (*Pongo abelii, Mus musculus, Rattus norvegicus, Gallus gallus, Xenopus tropicalis*), 92–98% of the entries have Swiss-Prot entry names identical with that of the best matching human entry (e.g. ALDOB_HUMAN, ALDOB_PONAB, ALDOB_MOUSE; ALDOB_RAT, ALDOB_CHICK). Since Swiss-Prot establishes orthology relationships by combining information from a variety of complementary sources (including scientific literature, sequence analysis tools, phylogenetic and comparative genomics databases such as Ensembl Compara, and other specialized databases such as species-specific collections [74], we have taken the identity of the Swiss-Prot entry name within this group as sufficient (but not necessary) evidence for 1:1 orthology. In the case of worm and fly a lower proportion of Swiss-Prot entries (42.7% and 48.1%, respectively) had entry names identical with those of the best matching human entry. Analysis of entries assigned to the same orthology groups but carrying entry names different from their human match by InParanoid [75,76], the P-POD: Princeton Protein Orthology Database [77,78] or TreeFam [31,79] confirmed that they also belong to the same orthology group. One major reason why orthologous Swiss-Prot entries do not have identical names is that in the case of 1:2, 1:3, 1:4 orthologs etc. the names of co-orthologs are distinguished by extra letters (e.g., DHSDB_DANRE *vs.* DHSD_HUMAN; SOBPA_DANRE *vs.* SOBP_HUMAN) or by extra numbers (e.g., PLOD3_HUMAN *vs.* PLOD_CAEEL; RYK_HUMAN *vs.* RYK2_DROME). Another major reason for the non-identity of the entry names of orthologs is that Swiss-Prot's apparent intention to give similar names to orthologs has not been fully applied (e.g., UNC6_CAEEL *vs.* NETA_DROME *vs.* NET1_HUMAN; LIN12_CAEEL *vs.* NOTCH_DROME *vs.* NOTC1_HUMAN). It must be emphasized that the majority of orthologous clusters contain multidomain proteins (*i.e.*, at least two PfamA domains) and that their orthology relationship was correctly determined at great evolutionary distances even in the case of orthologs with different domain architectures (e.g., A4_HUMAN NETR_MOUSE; see Figure S4), suggesting that correct orthology assignment by the triangulation method is relatively insensitive to changes in domain architecture. *vs.* A4_CAEEL, A4_DROME; MUSK_HUMAN *vs.* MUSK_CHICKEN; NETR_HUMAN *vs.* 3 **Datasets of Orthologous UniProtKB/TrEMBL, Refseq, EnsEMBL and NCBI's GNOMON Predicted Sequences** Orthology relationship between Swiss-Prot entries of *Homo sapiens* and UniProtKB/TrEMBL, Refseq, EnsEMBL and NCBI's GNOMON predicted sequences of other species were established in the same way as described above for comparison of Swiss-Prot entries: these sequences were blasted against human Swiss-Prot entries and sequences giving the best match (E value cut-off < 10^−5^) with the same human Swiss-Prot sequence were assigned to the same orthology cluster. The correspondence/equivalence between human Swiss-Prot entries and human UniProtKB/TrEMBL, Refseq, EnsEMBL and NCBI's GNOMON predicted sequences were established in a similar way.

Figure S1 Domain architectures of vertebrate agrins. Note that the DA of Swiss-Prot entries of rat and mouse agrin differ from those of chicken and human agrin since they represent different isoforms encoded by similar genes. Also note that the DA of chick and human agrin appear to be different because Pfam assigns equivalent domains (follistatin domains) to different domain families (Kazal_1 and Kazal_2) of the same domain clan (Kazal).

Figure S2 Correction of the sequence of rat DCLK1_RAT by the FixPred protocol. The DA of DCLK1_RAT was found to differ from those of DCLK1_MOUSE and DCLK1_HUMAN: whereas the latter contain two DCX and a Pkinase domain, the rat sequence lacks DCX domains. The sequence DCLK1_RAT_CORRECTED was predicted by the use of alternative gene models and is supported by ESTs FN798821, CF978300 and CB798849. (a) Comparison of the domain architecture of DCLK1_RAT with those of the correct DCLK1_HUMAN, DCLK1_MOUSE and DCLK1_RAT _CORRECTED sequences. (b) Alignment of the sequence of DCLK1_RAT with the correct DCLK_HUMAN, DCLK1_MOUSE and DCLK1_RAT_CORRECTED sequences.

Figure S3 Evidence that SYWM_CAEEL is mispredicted. The Swiss-Prot SYWM_CAEEL sequence arose by in silico fusion of the gene encoding the worm ortholog of PEX10 proteins and the worm ortholog of SYWM proteins. Note that no EST supports the existence of the fusion protein and that separate translation of these genes is supported by EST sequences BJ806113 of *Caenorhabditis elegans* and EST DR782673 of *Caenorhabditis remanei*. (a) Alignment of the mispredicted fusion sequence SYWM_CAEEL with its corrected constituents, PEX10_CAEEL and SYWM_CAEEL_CORRECTED; (b). Alignment of the FixPred predicted sequence of worm PEX10_CAEEL with orthologous PEX10 sequences; (C) Alignment of the FixPred corrected sequence SYWM_CAEEL_CORRECTED with orthologous SYWM sequences.

Figure S4 Examples of DA change during evolution of orthologs. (a) Comparison of the DA of MUSK_CHICK with those of MUSK_HUMAN, MUSK_MOUSE and MUSK_RAT; (b) Comparison of the DA of DCBD1_MOUSE and DCBD1_HUMAN; (c) Comparison of the DA of NETR_HUMAN and NETR_MOUSE; (d) Comparison of the DA of A4_HUMAN, A4_CAEEL and A4_DROME.

Figure S5 Misprediction of the sequence of the FZD8 protein of *Gallus gallus* by GNOMON. The DA of the GNOMON-predicted sequence of the FZD8 ortholog from *Gallus gallus* (XP_426568) was found to differ from that of FZD8_HUMAN: whereas the latter contains an Fz and a Frizzled domain (as well as a signal peptide), the ortholog of *Gallus gallus* lacks the Fz domain and the Frizzled domain is N-terminally truncated. (a) Comparison of the DAs of FZD8_HUMAN and XP_426568; (b) Alignment of the sequences of FZD8_HUMAN, FZD8_MOUSE FZD8_XENLA, FZD5_XENLA and XP_426568.

Figure S6 Correction of the sequence of the Kremen 1 protein of *Xenopus tropicalis* with the FixPred protocol. The DA of the Refseq ortholog of kremen 1 from *Xenopus tropicalis* (NP_001116927) was found to differ from that of KREM1_HUMAN: whereas the latter contains a Kringle, a WSC and a CUB domain (as well as a signal peptide and a transmembrane segment), the ortholog of *Xenopus tropicalis* lacks the kringle domain. The sequence ‘KREM1_XENTR_CORRECTED’ was predicted by the use of alternative gene models and is supported by ESTs DT392278 and EL798390. (a) Comparison of the DAs of KREM1_HUMAN, NP_001116927 and KREM1_XENTR_CORRECTED; (b) Alignment of the sequences of KREM1 proteins from human, mouse, rat and the frog *Xenopus laevis* with NP_001116927 and the corrected sequence, KREM1_XENTR_CORRECTED.

Figure S7 Correction of the sequence of the Kremen 2 protein of *Xenopus tropicalis* with the FixPred protocol. The DA of Refseq ortholog of kremen 2 from *Xenopus tropicalis* (NP_001072931) was found to differ from those of KREM1_HUMAN, KREM1_MOUSE, KREM1_RAT and the ortholog from *Xenopus laevis*, Q8AXX3_XENLA: whereas the latter contain a Kringle, a WSC and a CUB domain (as well as a signal peptide and a transmembrane segment), the ortholog of *Xenopus tropicalis* lacks the CUB domain. The sequence ‘KREM2_XENTR_CORRECTED’ was predicted by the use of alternative gene models and is supported by ESTs DT425049 and DT425818. (a) Comparison of the DAs of KREM2_HUMAN, Q8AXX3_XENLA, NP_001072931 and KREM1_XENTR_CORRECTED; (b) Alignment of the sequence of Q8AXX3_XENLA with NP_001072931 and the corrected sequence, KREM1_XENTR_CORRECTED.

Figure S8 Correction of the sequence of the XP_416936 protein of *Gallus gallus* with the FixPred protocol. The DA of the GNOMON predicted protein XP_416936 was found to differ from those of GAS6_MOUSE, GAS6_RAT, GAS6_HUMAN: whereas the latter contain a signal peptide, a Gla, three EGF_CA, a Laminin_G_1 and a Laminin_G_2 domain, XP_416936 lacks the N-terminal signal peptide and Gal domain. The sequence XP_416936_CORRECTED was predicted by the use of ESTs CD217792, BM439645 and BU115578. (a) Comparison of the DAs of XP_416936, some of the four EGF_CA domains of GAS6 proteins are detected with E-values >0.0001 and are not represented in the DA images generated by Pfam. (b) Alignment of the sequences of XP_416936, XP_416936_CORRECTED with those of GAS6_MOUSE, GAS6_RAT and GAS6_HUMAN. Note that XP_416936_CORRECTED with those of GAS6_MOUSE, GAS6_RAT and GAS6_HUMAN.

**Table S1:** Times of divergence of *Homo sapiens* from the lineages of the species analyzed. In our analyses we used average values determined for all genes taken from the homepage of TimeTree [44].

**Taxa compared**	**Divergence time (Mya)**			**Species compared***
	**Simple Average**	**Weighted Average**	**Expert**	
Homo/Pongo	15.96	15.48		*Homo-Pongo*
Primates/Glires	94.72	103.74	91	*Homo-Mus*
Mammalia/Sauropsida	274.80	324.81	325	*Homo-Gallus*
Amniota/Amphibia	389.66	360.50	361	*Homo-Xenopus*
Sarcopterygii/Actinopterygii	444.25	454.94	455	*Homo-Danio*
Deuterostomia/Protostomia	826.36	980.12	910	*Homo-Drosophila*
Coelomata/Pseudocoelomata	993.57	867.44	728	*Homo-Caenorhabditis*

* The species are listed in the order of increasing evolutionary distance from *Homo sapiens*.

Table S2 Proportion of orthologous sequences of Metazoa that differ in 1, 2, 3, or ≥4 domains from their human Swiss-Prot ortholog.

**Table:**

**Table S2/A.** Proportion of orthologous Swiss-Prot sequences of Metazoa that differ in 1, 2, 3, or ≥4 domains from their human Swiss-Prot ortholog.				
**Species**	**Number of Domains Distinguishing Das ***			
	**N = 1**	**N = 2**	**N = 3**	**N≥4**
*Pongo abelii*	66,66	0,00	0,00	33,33
*Mus musculus*	73,70	11,29	5,07	9,91
*Gallus gallus*	65,70	9,14	5,71	19,42
*Xenopus tropicalis*	65,60	34,38	0,00	0,00
*Danio rerio*	74,30	12,39	5,31	7,96
*Drosophila melanogaster*	71,00	20,39	3,95	4,61
*Caenorhabditis elegans*	60,80	27,11	3,61	8,43
*The numbers in the different categories represent the percent of total DA differences.				

**Table S2/B.** Proportion of orthologous/equivalent TrEMBL sequences of Metazoa that differ in 1, 2, 3, or ≥4 domains from their human Swiss-Prot ortholog.				
**Species**	**Number of Domains Distinguishing DAs***			
	**N = 1**	**N = 2**	**N = 3**	**N≥4**
*Homo sapiens*	62,40	17,19	8,10	4,27
*Mus musculus*	57,94	19,03	8,39	4,59
*Gallus gallus*	59,83	20,17	5,45	3,20
*Xenopus tropicalis*	56,20	17,95	9,74	6,68
*Danio rerio*	58,86	19,38	9,24	4,35
*Drosophila melanogaster*	60,78	18,44	8,86	4,54
*Caenorhabditis elegans*	59,91	18,26	8,52	4,23
* The numbers in the different categories represent the percent of total DA differences.				

**Table S2/C.** Proportion of RefSeq sequences of Metazoa that differ in 1, 2, 3, or ≥4 domains from their human Swiss-Prot ortholog/equivalent				
**Species**	**Number of Domains Distinguishing DAs***			
	**N = 1**	**N = 2**	**N = 3**	**N≥4**
*Homo sapiens*	69,23	13,77	2,02	14,97
*Mus musculus*	74,42	12,43	4,48	8,67
*Gallus gallus*	67,74	17,01	6,38	8,87
*Xenopus tropicalis*	65,6	14,86	6,43	13,12
*Danio rerio*	65,11	18,55	6,55	9,79
*Drosophila melanogaster*	65,96	16,86	6,72	10,45
*Caenorhabditis elegans*	64,76	17,51	6,76	10,97
* The numbers in the different categories represent the percent of total DA differences.				

**Table S2/D.** Proportion of NCBI's GNOMON predicted sequences of Metazoa that differ in 1, 2, 3, or ≥4 domains from their human Swiss-Prot ortholog/equivalent.				
**Species**	**Number of Domains Distinguishing DAs***			
	**N = 1**	**N = 2**	**N = 3**	**N≥4**
*Homo sapiens*	48,65	18,43	13,51	19,41
*Mus musculus*	55,55	16,24	6,50	21,71
*Gallus gallus*	61,21	19,20	6,51	11,55
*Danio rerio*	60,63	18,49	7,68	13,20
*Drosophila pseudoobscura*	65,47	17,64	6,89	10,00
*Caenorhabditis briggsae*	66,68	16,31	6,41	10,59
* The numbers in the different categories represent the percent of total DA differences.				

**Table S2/E.** Proportion of EnsEMBLsequences of Metazoa that differ in 1, 2, 3, or ≥4 domains from their human Swiss-Prot ortholog/equivalent.				
**Species**	**Number of Domains Distinguishing DAs***			
	**N = 1**	**N = 2**	**N = 3**	**N≥4**
*Homo sapiens*	88,08	8,85	3,08	0,00
*Mus musculus*	66,30	15,93	5,27	12,50
*Gallus gallus*	67,28	16,33	6,90	9,49
*Xenopus tropicalis*	61,61	18,86	6,15	13,38
*Danio rerio*	65,83	18,16	5,97	10,04
*Drosophila melanogaster*	63,93	18,83	7,30	9,95
*Caenorhabditis elegans*	62,84	19,28	6,78	11,11

* The numbers in the different categories represent the percent of total DA differences.
