# Correction: Nagy, A., *et al.* Reassessing Domain Architecture Evolution of Metazoan Proteins: Major Impact of Gene Prediction Errors

**DOI:** 10.3390/genes2030599

**Published:** 2011-08-16

**Authors:** Alinda Nagy, György Szláma, Eszter Szarka, Mária Trexler, László Bányai, László Patthy

**Affiliations:** Institute of Enzymology, Biological Research Center, Hungarian Academy of Sciences, H-1113 Budapest, Hungary; E-Mails: nagya@enzim.hu (A.N.); szlama@enzim.hu (G.S.); szarka@enzim.hu (E.S.); trexler@enzim.hu (M.T.); banyai@enzim.hu (L.B.)

We found some errors in the published versions of Figure S2, Figure S3 and Figure S8 of our paper [1]. The correct Figures are presented below.

**Figure S2:**
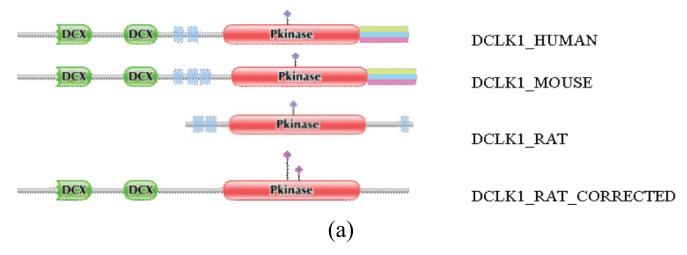

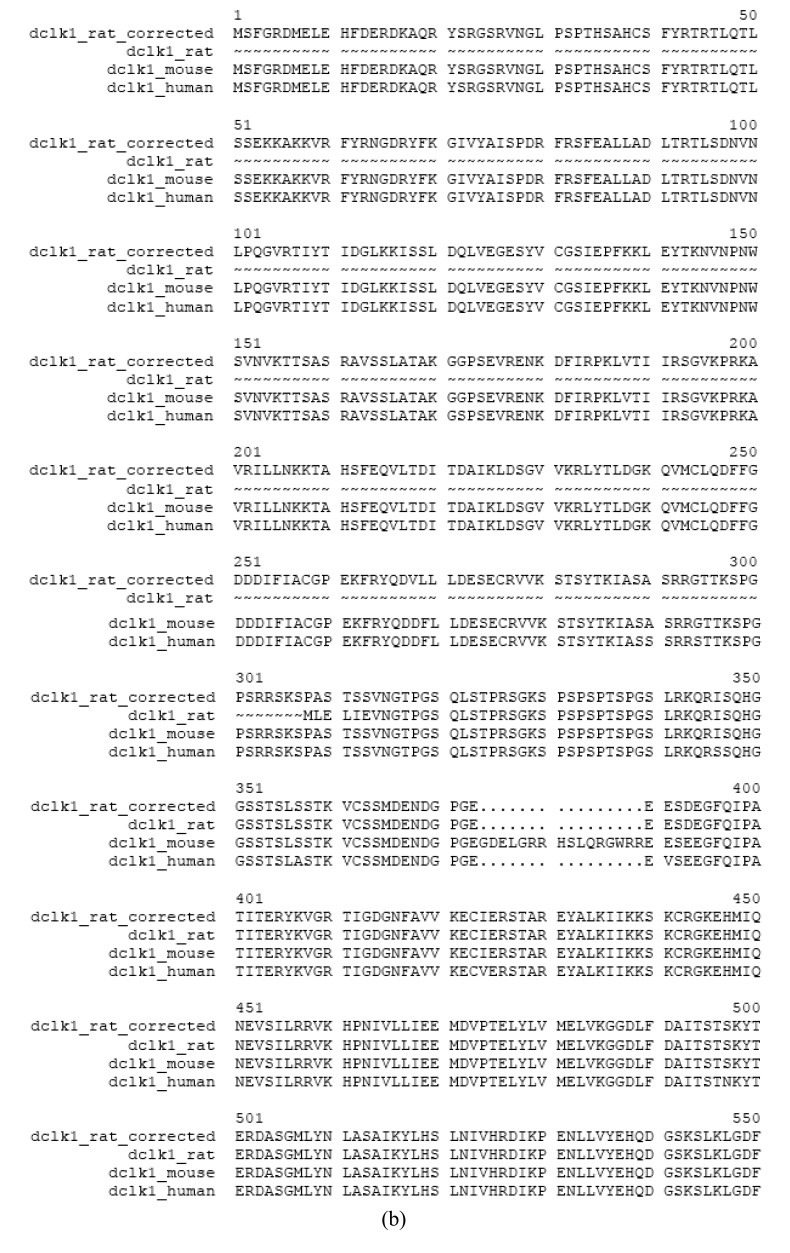

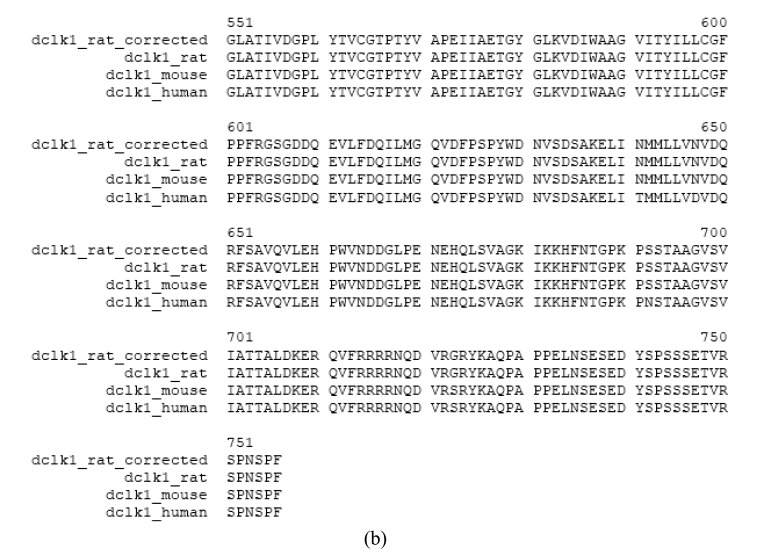
Correction of the sequence of rat DCLK1_RAT by the FixPred protocol. The DA of DCLK1_RAT was found to differ from those of DCLK1_MOUSE and DCLK1_HUMAN: whereas the latter contain two DCX and a Pkinase domain, the rat sequence lacks DCX domains. The sequence DCLK1_RAT_CORRECTED was predicted by the use of alternative gene models and is supported by ESTs FN798821, CF978300 and CB798849. (a) Comparison of the domain architecture of DCLK1_RAT with those of the correct DCLK1_HUMAN, DCLK1_MOUSE and DCLK1_RAT_CORRECTED sequences. (b) Alignment of the sequence of DCLK1_RAT with the correct DCLK_HUMAN, DCLK1_MOUSE and DCLK1_RAT_CORRECTED sequences.

**Figure S3:**
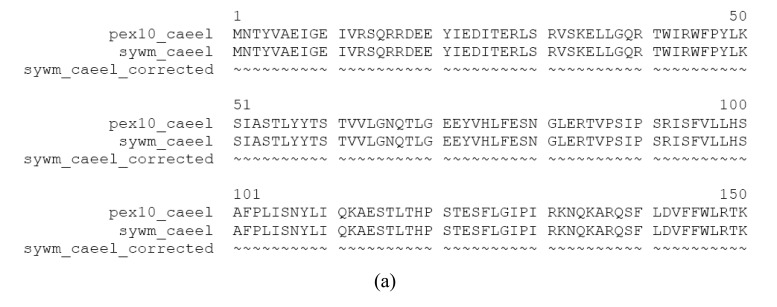

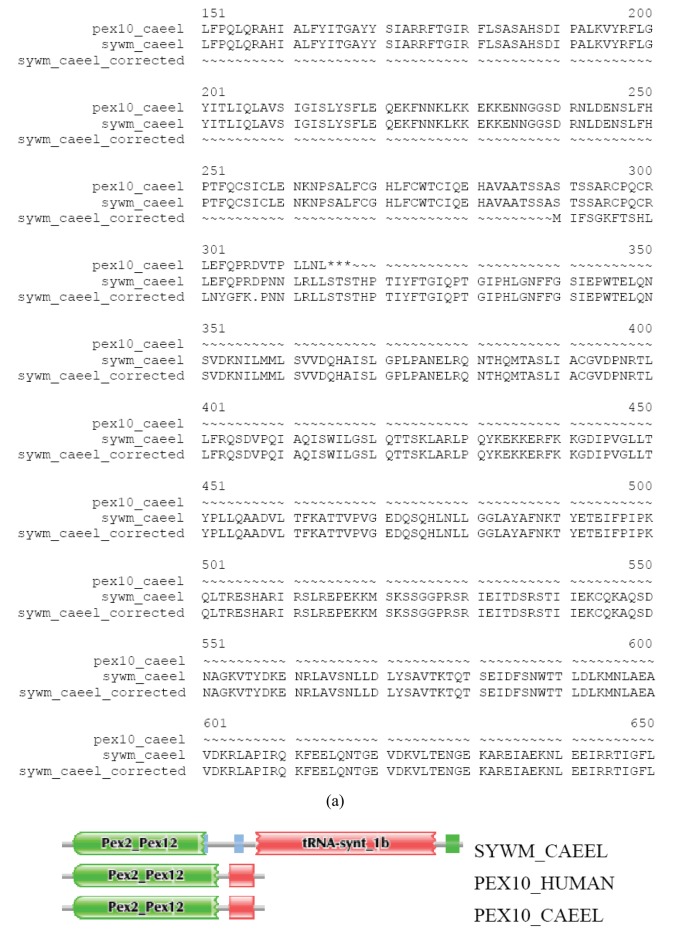

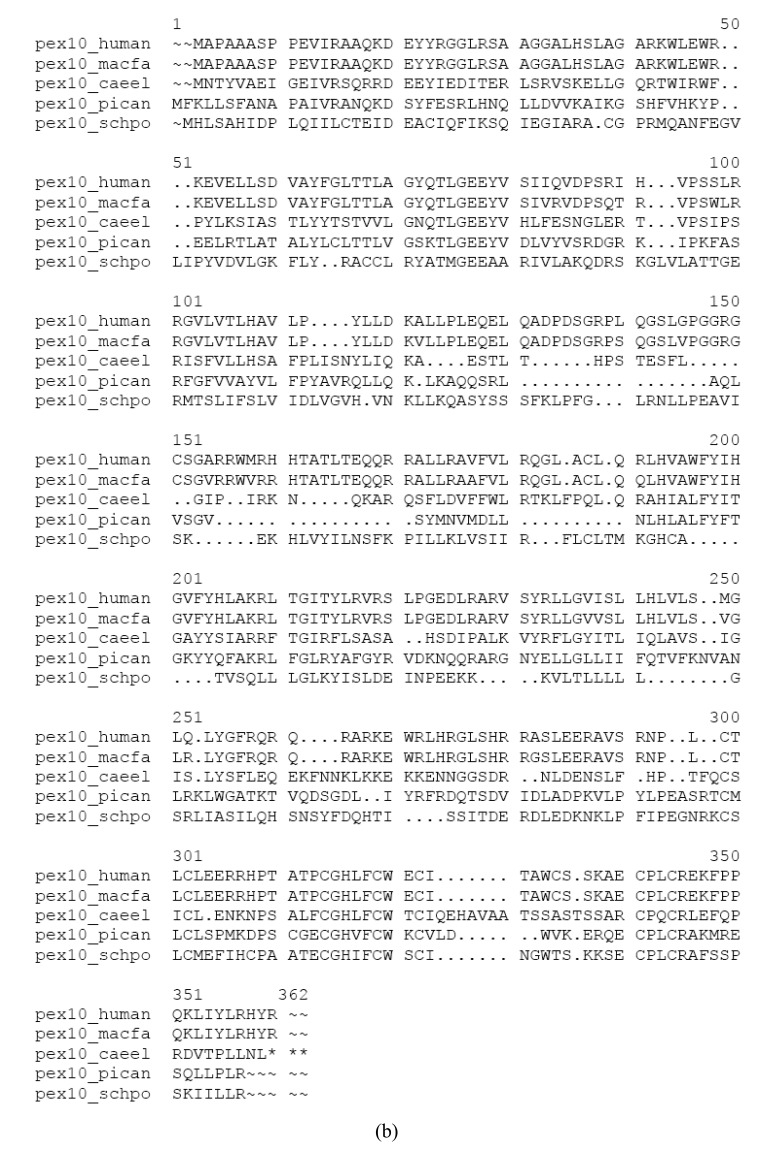

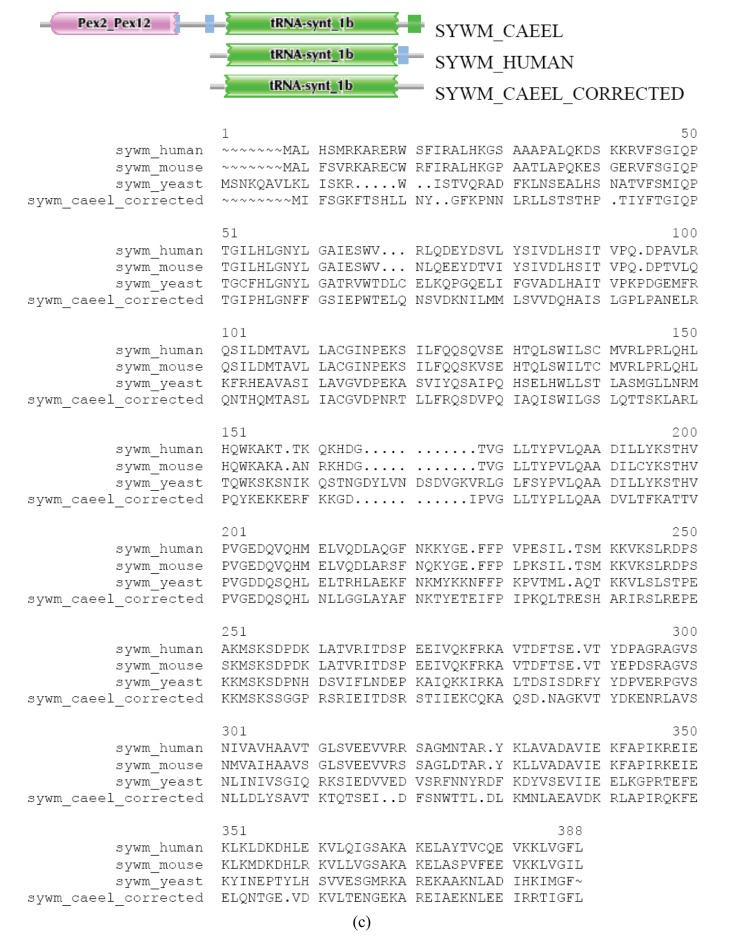
Evidence that SYWM_CAEEL is mispredicted. The Swiss-Prot SYWM_CAEEL sequence arose by *in silico* fusion of the gene encoding the worm ortholog of PEX10 proteins and the worm ortholog of SYWM proteins. Note that no EST supports the existence of the fusion protein and that separate translation of these genes is supported by EST sequences BJ806113 of *Caenorhabditis elegans* and EST DR782673 of *Caenorhabditis remanei*. (a) Alignment of the mispredicted fusion sequence SYWM_CAEEL with its corrected constituents, PEX10_CAEEL and SYWM_CAEEL_CORRECTED; (b). Alignment of the FixPred predicted sequence of worm PEX10_CAEEL with orthologous PEX10 sequences; (c) Alignment of the FixPred corrected sequence SYWM_CAEEL_CORRECTED with orthologous SYWM sequences.

**Figure S8:**
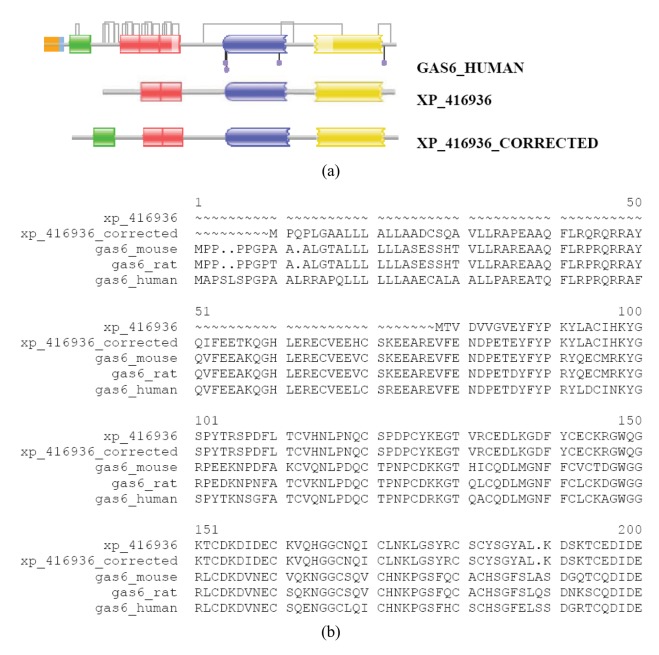

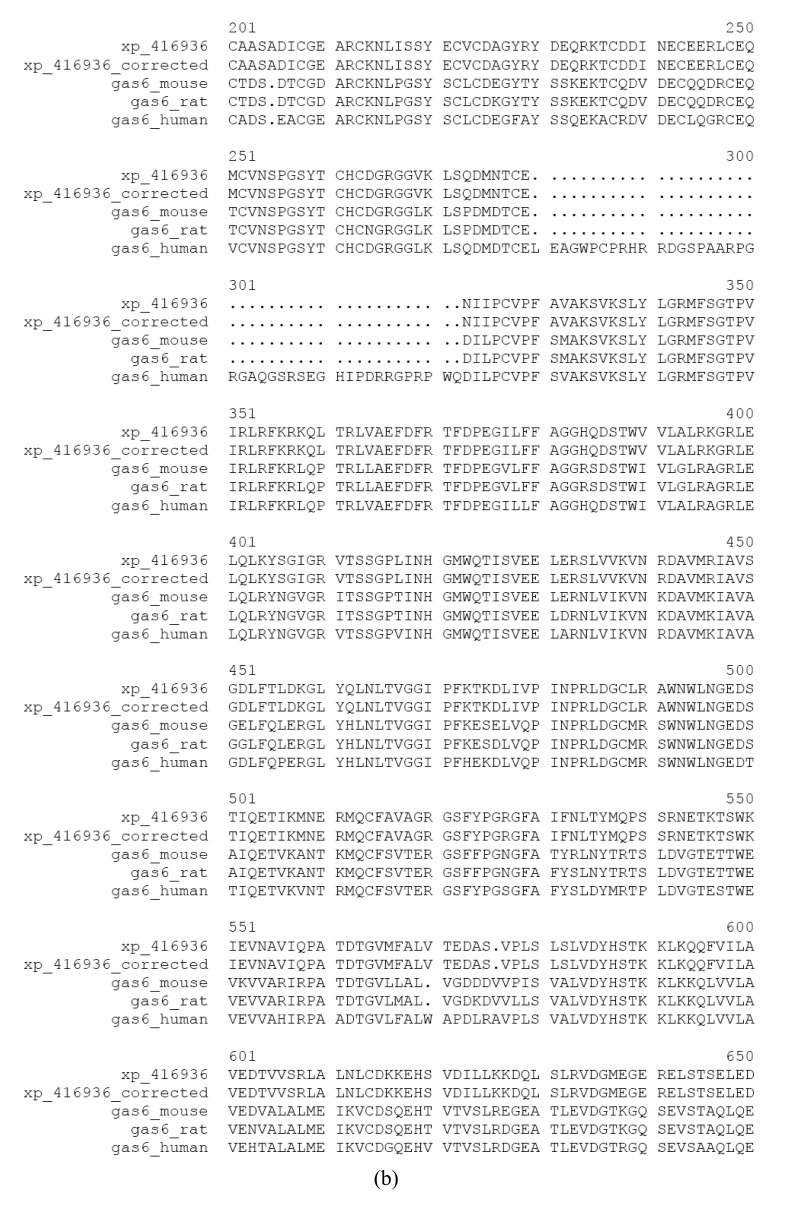

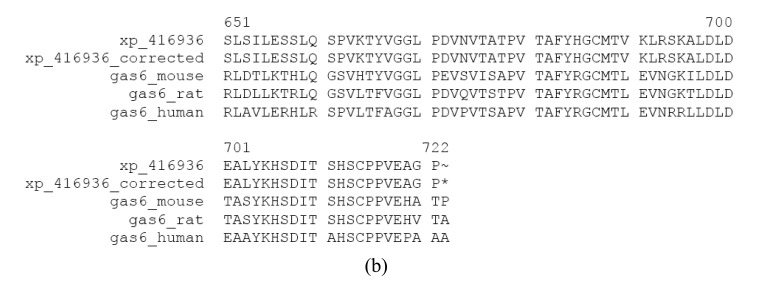
Correction of the sequence of the XP_416936 protein of *Gallus gallus* with the FixPred protocol. The DA of the GNOMON predicted protein XP_416936 was found to differ from those of GAS6_MOUSE, GAS6_RAT, GAS6_HUMAN: whereas the latter contain a signal peptide, a Gla, three EGF_CA, a Laminin_G_1 and a Laminin_G_2 domain, XP_416936 lacks the N-terminal signal peptide and Gla domain. The sequence XP_416936_CORRECTED was predicted by the use of ESTs CD217792, BM439645 and BU115578. (a) Comparison of the DAs of XP_416936, XP_416936_CORRECTED with those of GAS6_MOUSE, GAS6_RAT and GAS6_HUMAN. Note that some of the four EGF_CA domains of GAS6 proteins are detected with E-values >0.0001 and are not represented in the DA images generated by Pfam. (b) Alignment of the sequences of XP_416936, XP_416936_CORRECTED with those of GAS6_MOUSE, GAS6_RAT and GAS6_HUMAN.

We apologize for any inconvenience caused to the readers.
