# Supplementary figures and images for: Reassessing Domain Architecture Evolution of Metazoan Proteins: Major Impact of Gene Prediction Errors
Source: Genes (Basel). 2011 Jul 13;2(3):449–501. doi: 10.3390/genes2030449 (PMC3927609; doi:10.3390/genes2030449)

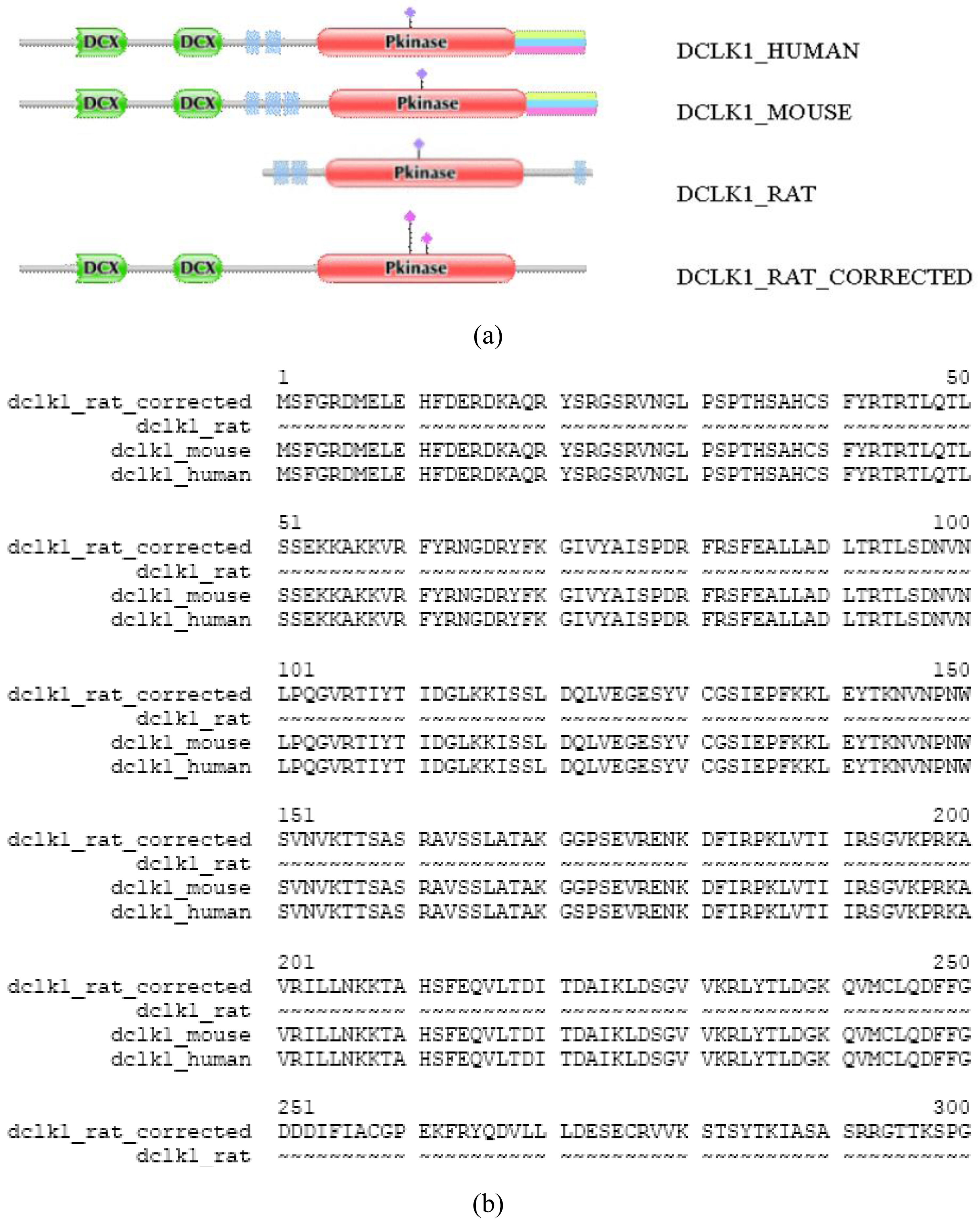

Supplement: Figure S2 — Correction of the sequence of rat DCLK1_RAT by the FixPred protocol. The DA of DCLK1_RAT was found to differ from those of DCLK1_MOUSE and DCLK1_HUMAN: whereas the latter contain two DCX and a Pkinase domain, the rat sequence lacks DCX domains. The sequence DCLK1_RAT_CORRECTED was predicted by the use of alternative gene models and is supported by ESTs FN798821, CF978300 and CB798849. (a) Comparison of the domain architecture of DCLK1_RAT with those of the correct DCLK1_HUMAN, DCLK1_MOUSE and DCLK1_RAT _CORRECTED sequences. (b) Alignment of the sequence of DCLK1_RAT with the correct DCLK_HUMAN, DCLK1_MOUSE and DCLK1_RAT_CORRECTED sequences. [file genes-02-00449f4.tif]

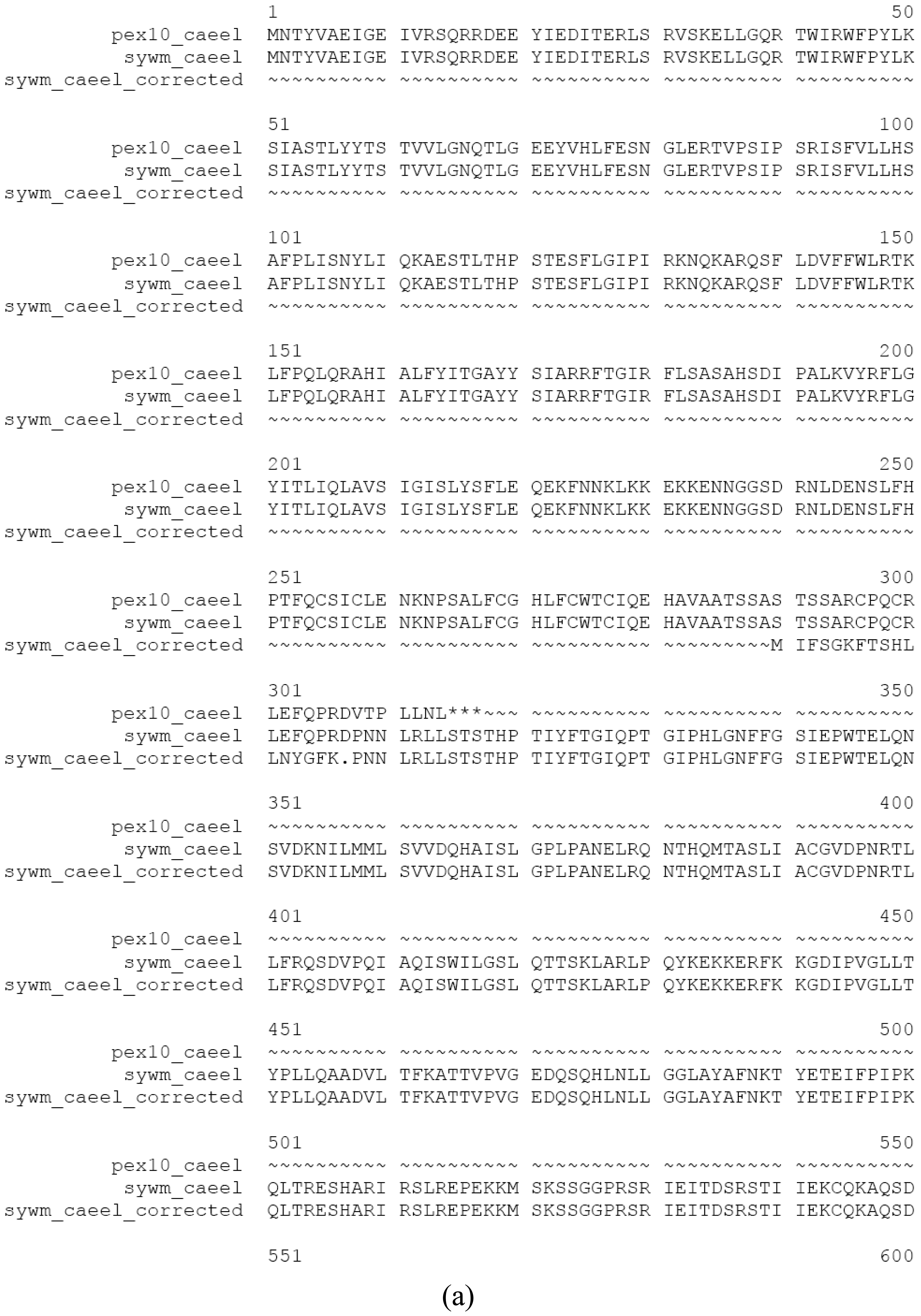

Supplement: Figure S3 — Evidence that SYWM_CAEEL is mispredicted. The Swiss-Prot SYWM_CAEEL sequence arose by in silico fusion of the gene encoding the worm ortholog of PEX10 proteins and the worm ortholog of SYWM proteins. Note that no EST supports the existence of the fusion protein and that separate translation of these genes is supported by EST sequences BJ806113 of Caenorhabditis elegans and EST DR782673 of Caenorhabditis remanei. (a) Alignment of the mispredicted fusion sequence SYWM_CAEEL with its corrected constituents, PEX10_CAEEL and SYWM_CAEEL_CORRECTED; (b). Alignment of the FixPred predicted sequence of worm PEX10_CAEEL with orthologous PEX10 sequences; (C) Alignment of the FixPred corrected sequence SYWM_CAEEL_CORRECTED with orthologous SYWM sequences. [file genes-02-00449f5a.tif]

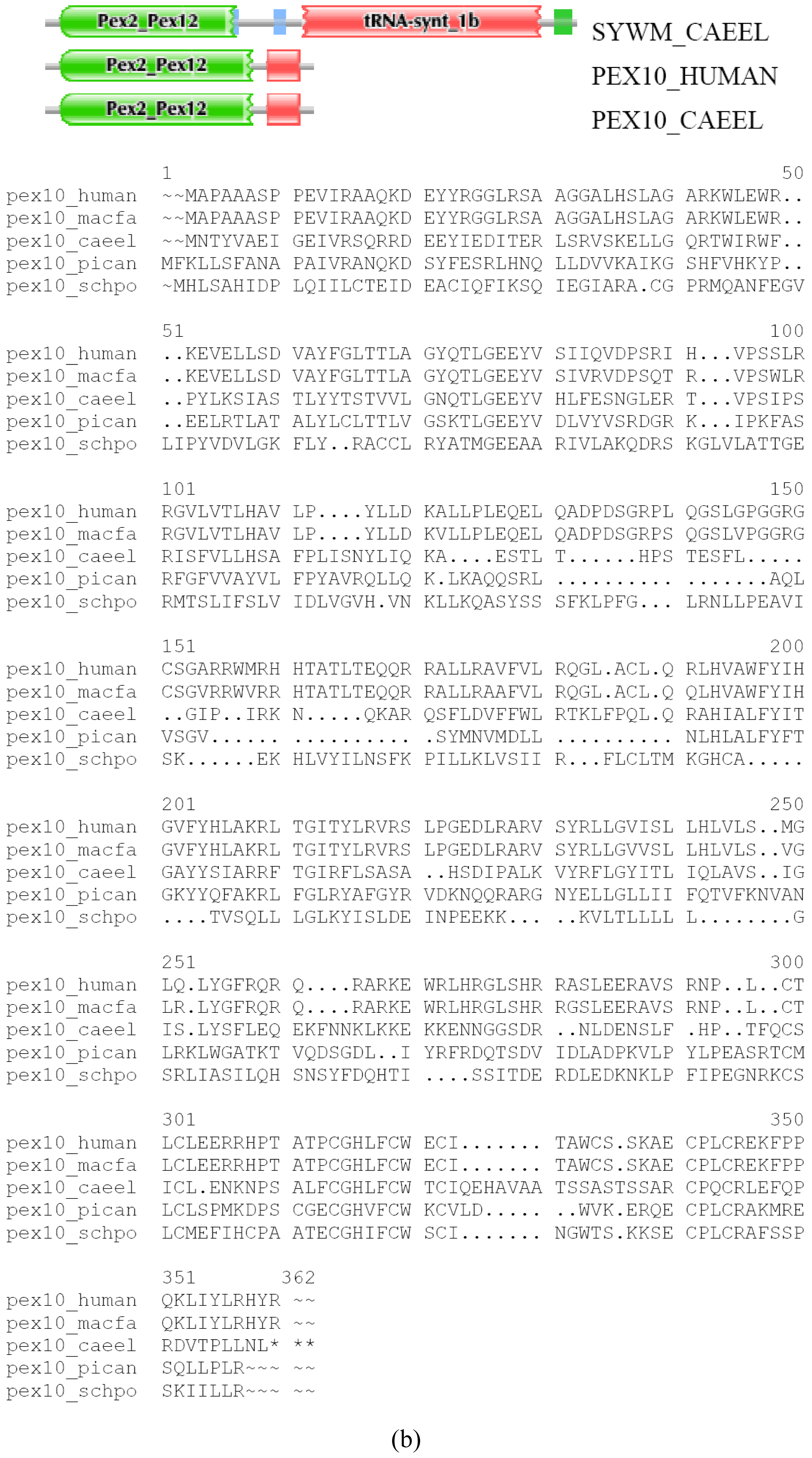

Supplement: Figure S3 — Evidence that SYWM_CAEEL is mispredicted. The Swiss-Prot SYWM_CAEEL sequence arose by in silico fusion of the gene encoding the worm ortholog of PEX10 proteins and the worm ortholog of SYWM proteins. Note that no EST supports the existence of the fusion protein and that separate translation of these genes is supported by EST sequences BJ806113 of Caenorhabditis elegans and EST DR782673 of Caenorhabditis remanei. (a) Alignment of the mispredicted fusion sequence SYWM_CAEEL with its corrected constituents, PEX10_CAEEL and SYWM_CAEEL_CORRECTED; (b). Alignment of the FixPred predicted sequence of worm PEX10_CAEEL with orthologous PEX10 sequences; (C) Alignment of the FixPred corrected sequence SYWM_CAEEL_CORRECTED with orthologous SYWM sequences. [file genes-02-00449f5b.tif]

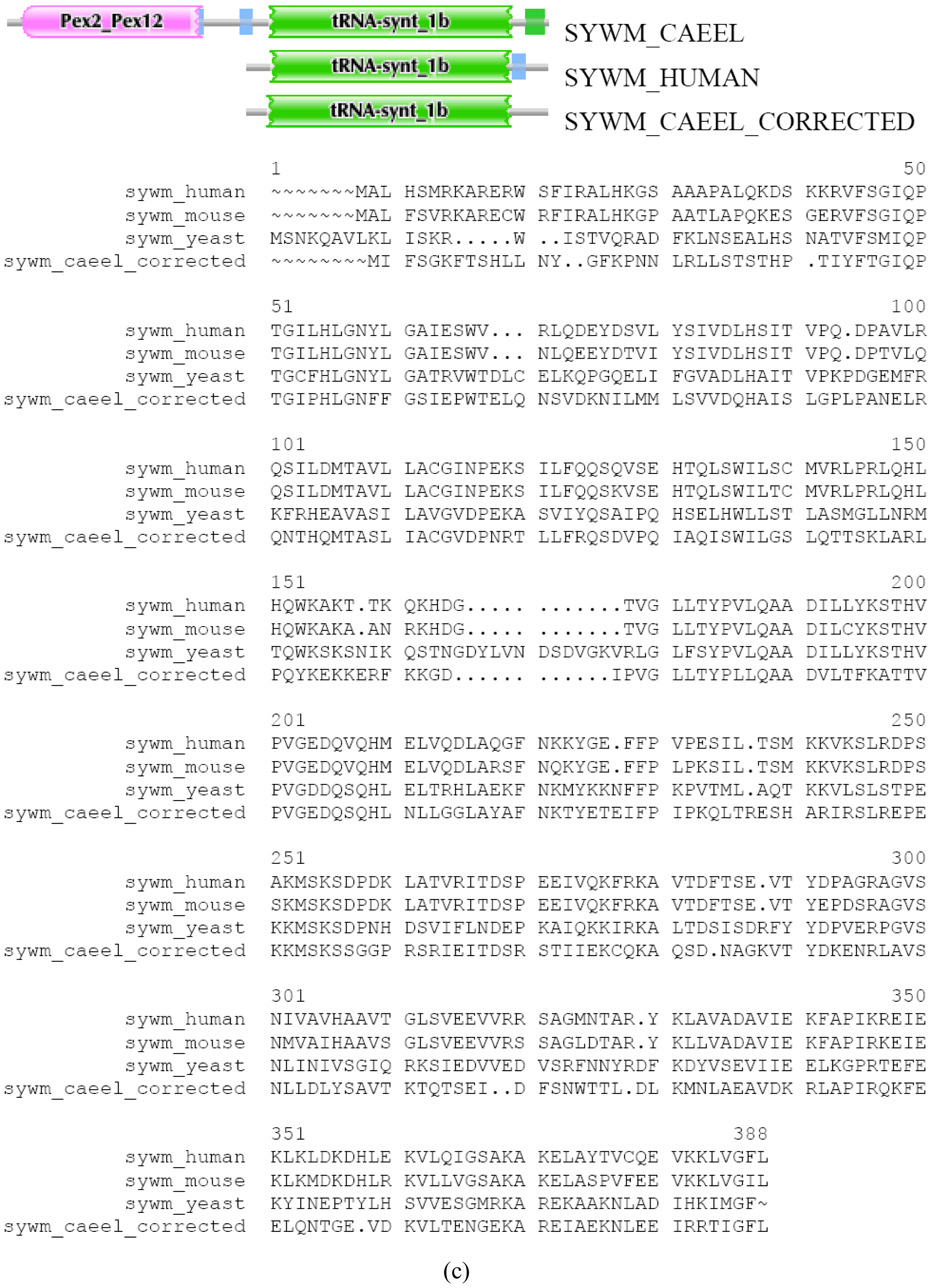

Supplement: Figure S3 — Evidence that SYWM_CAEEL is mispredicted. The Swiss-Prot SYWM_CAEEL sequence arose by in silico fusion of the gene encoding the worm ortholog of PEX10 proteins and the worm ortholog of SYWM proteins. Note that no EST supports the existence of the fusion protein and that separate translation of these genes is supported by EST sequences BJ806113 of Caenorhabditis elegans and EST DR782673 of Caenorhabditis remanei. (a) Alignment of the mispredicted fusion sequence SYWM_CAEEL with its corrected constituents, PEX10_CAEEL and SYWM_CAEEL_CORRECTED; (b). Alignment of the FixPred predicted sequence of worm PEX10_CAEEL with orthologous PEX10 sequences; (C) Alignment of the FixPred corrected sequence SYWM_CAEEL_CORRECTED with orthologous SYWM sequences. [file genes-02-00449f5c.tif]

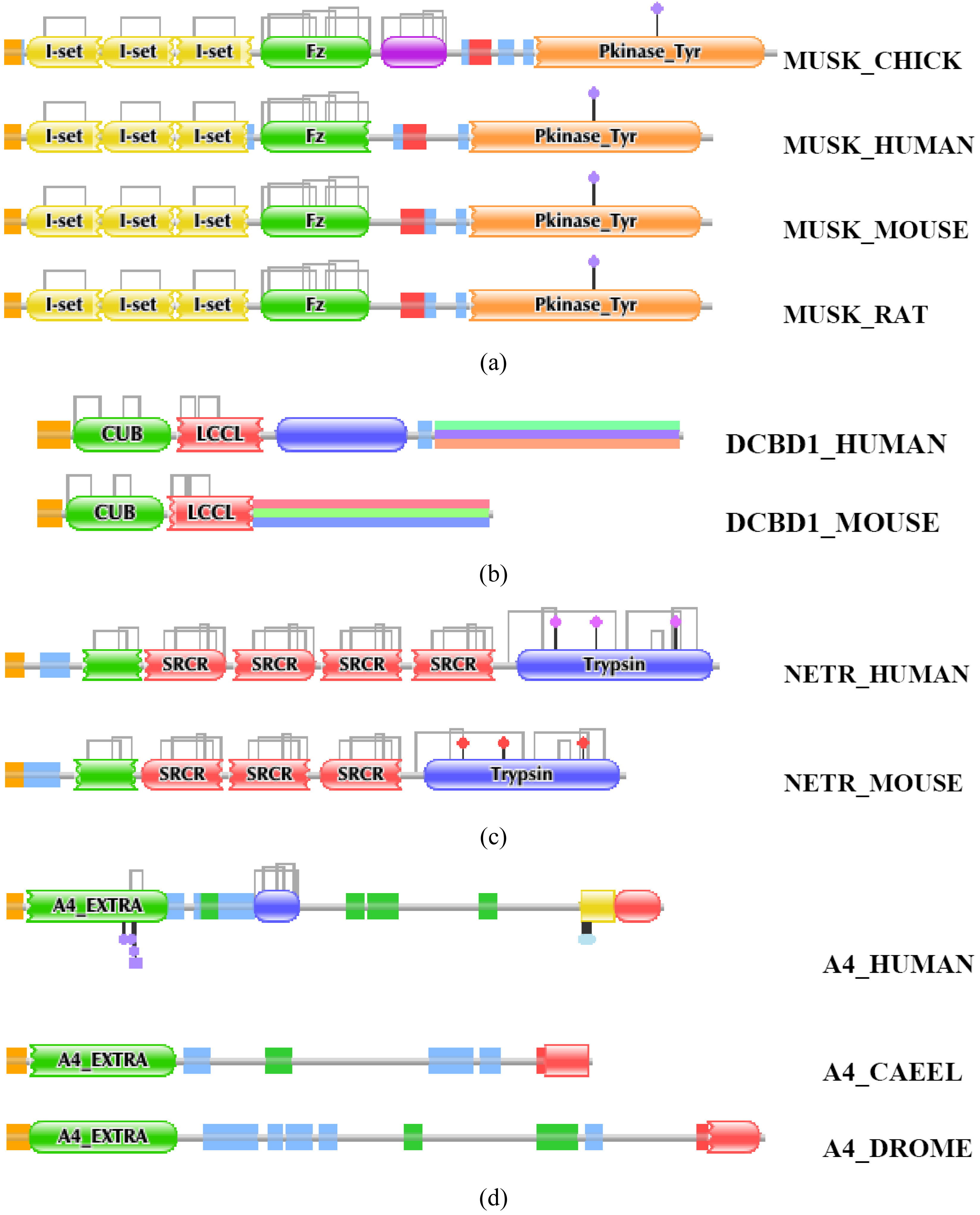

Supplement: Figure S4 — Examples of DA change during evolution of orthologs. (a) Comparison of the DA of MUSK_CHICK with those of MUSK_HUMAN, MUSK_MOUSE and MUSK_RAT; (b) Comparison of the DA of DCBD1_MOUSE and DCBD1_HUMAN; (c) Comparison of the DA of NETR_HUMAN and NETR_MOUSE; (d) Comparison of the DA of A4_HUMAN, A4_CAEEL and A4_DROME. [file genes-02-00449f6.tif]

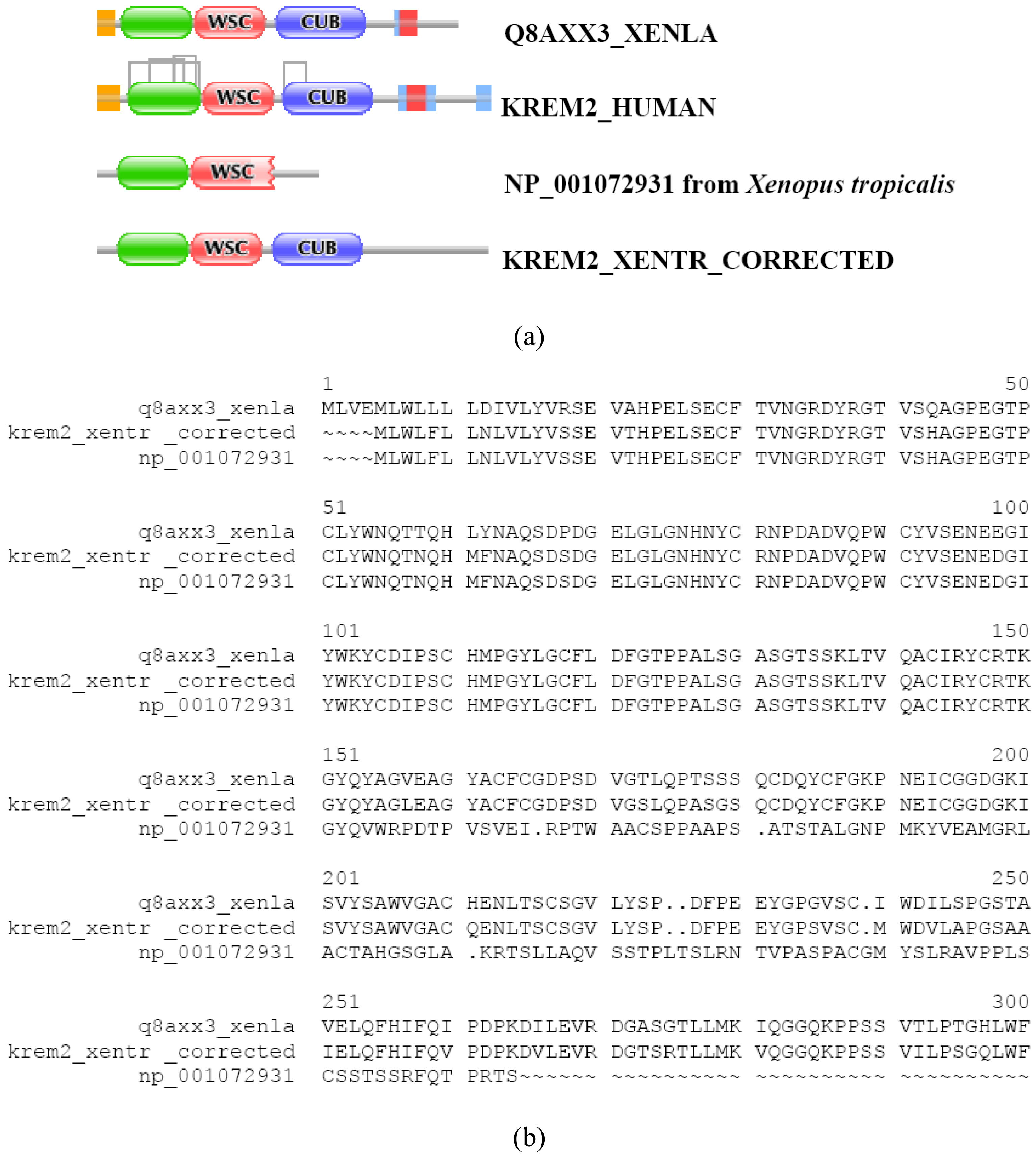

Supplement: Figure S5 — Misprediction of the sequence of the FZD8 protein of Gallus gallus by GNOMON. The DA of the GNOMON-predicted sequence of the FZD8 ortholog from Gallus gallus (XP_426568) was found to differ from that of FZD8_HUMAN: whereas the latter contains an Fz and a Frizzled domain (as well as a signal peptide), the ortholog of Gallus gallus lacks the Fz domain and the Frizzled domain is N-terminally truncated. (a) Comparison of the DAs of FZD8_HUMAN and XP_426568; (b) Alignment of the sequences of FZD8_HUMAN, FZD8_MOUSE FZD8_XENLA, FZD5_XENLA and XP_426568. [file genes-02-00449f7a.tif]

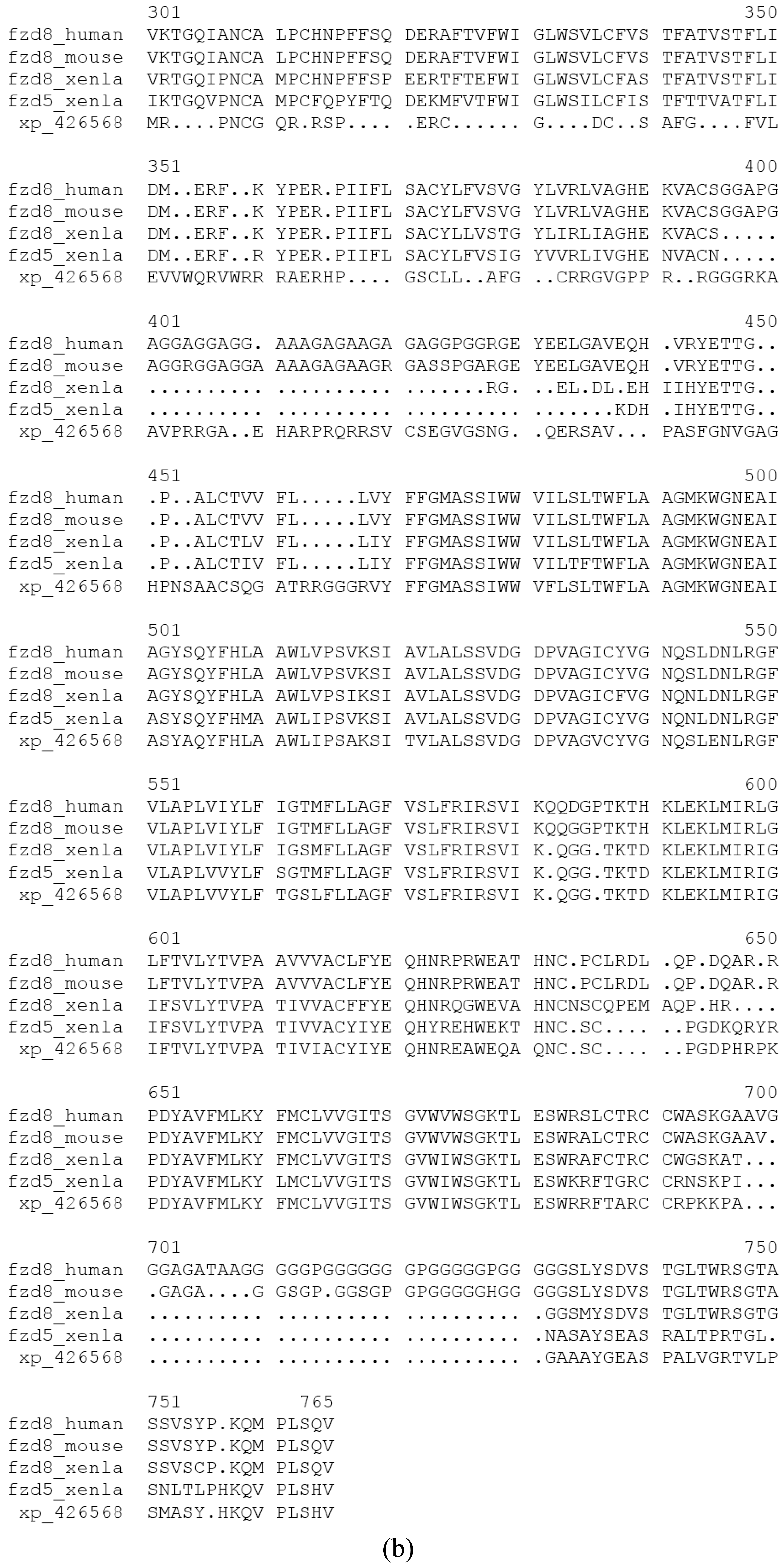

Supplement: Figure S5 — Misprediction of the sequence of the FZD8 protein of Gallus gallus by GNOMON. The DA of the GNOMON-predicted sequence of the FZD8 ortholog from Gallus gallus (XP_426568) was found to differ from that of FZD8_HUMAN: whereas the latter contains an Fz and a Frizzled domain (as well as a signal peptide), the ortholog of Gallus gallus lacks the Fz domain and the Frizzled domain is N-terminally truncated. (a) Comparison of the DAs of FZD8_HUMAN and XP_426568; (b) Alignment of the sequences of FZD8_HUMAN, FZD8_MOUSE FZD8_XENLA, FZD5_XENLA and XP_426568. [file genes-02-00449f7b.tif]

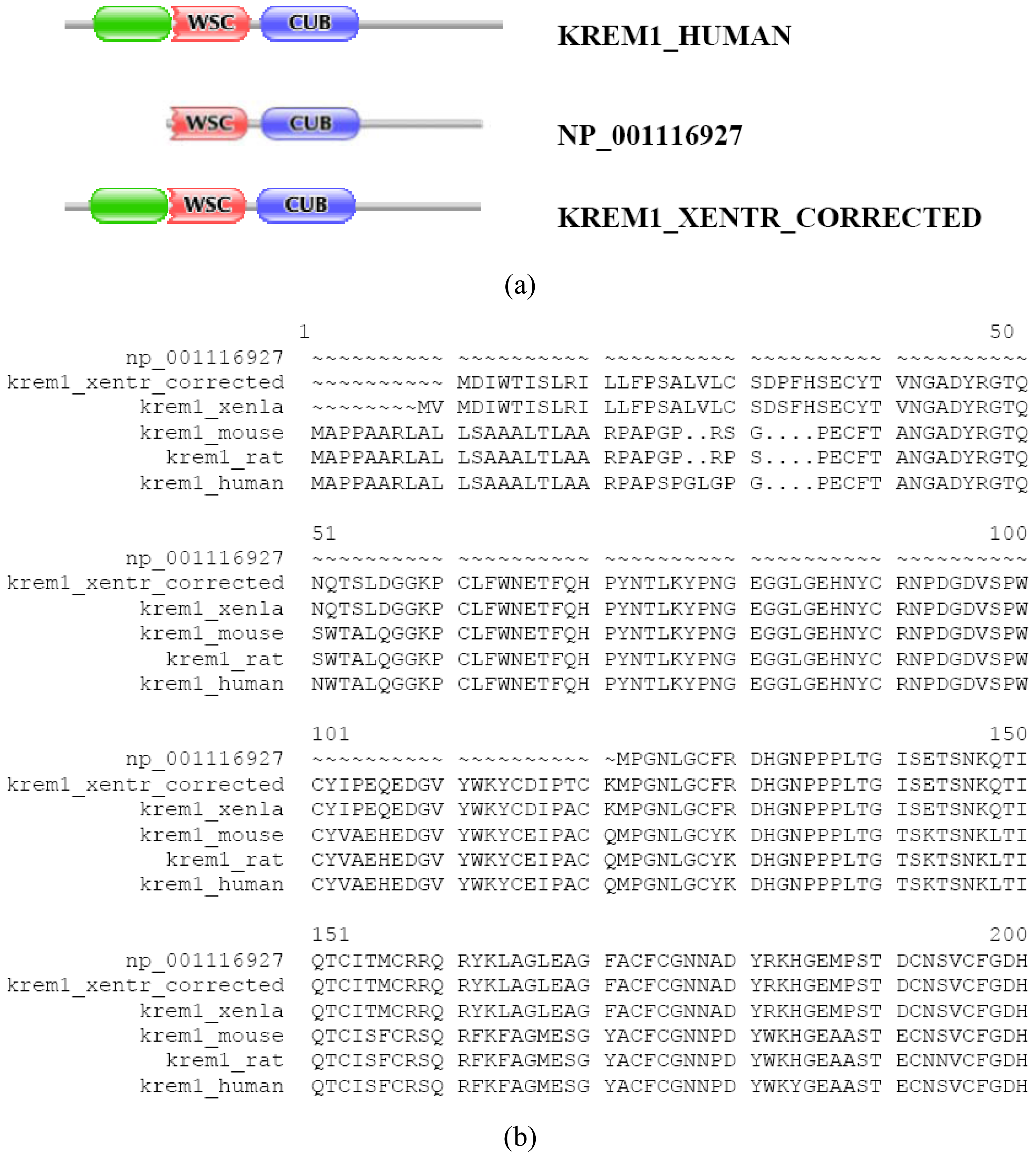

Supplement: Figure S6 — Correction of the sequence of the Kremen 1 protein of Xenopus tropicalis with the FixPred protocol. The DA of the Refseq ortholog of kremen 1 from Xenopus tropicalis (NP_001116927) was found to differ from that of KREM1_HUMAN: whereas the latter contains a Kringle, a WSC and a CUB domain (as well as a signal peptide and a transmembrane segment), the ortholog of Xenopus tropicalis lacks the kringle domain. The sequence ‘KREM1_XENTR_CORRECTED’ was predicted by the use of alternative gene models and is supported by ESTs DT392278 and EL798390. (a) Comparison of the DAs of KREM1_HUMAN, NP_001116927 and KREM1_XENTR_CORRECTED; (b) Alignment of the sequences of KREM1 proteins from human, mouse, rat and the frog Xenopus laevis with NP_001116927 and the corrected sequence, KREM1_XENTR_CORRECTED. [file genes-02-00449f8a.tif]

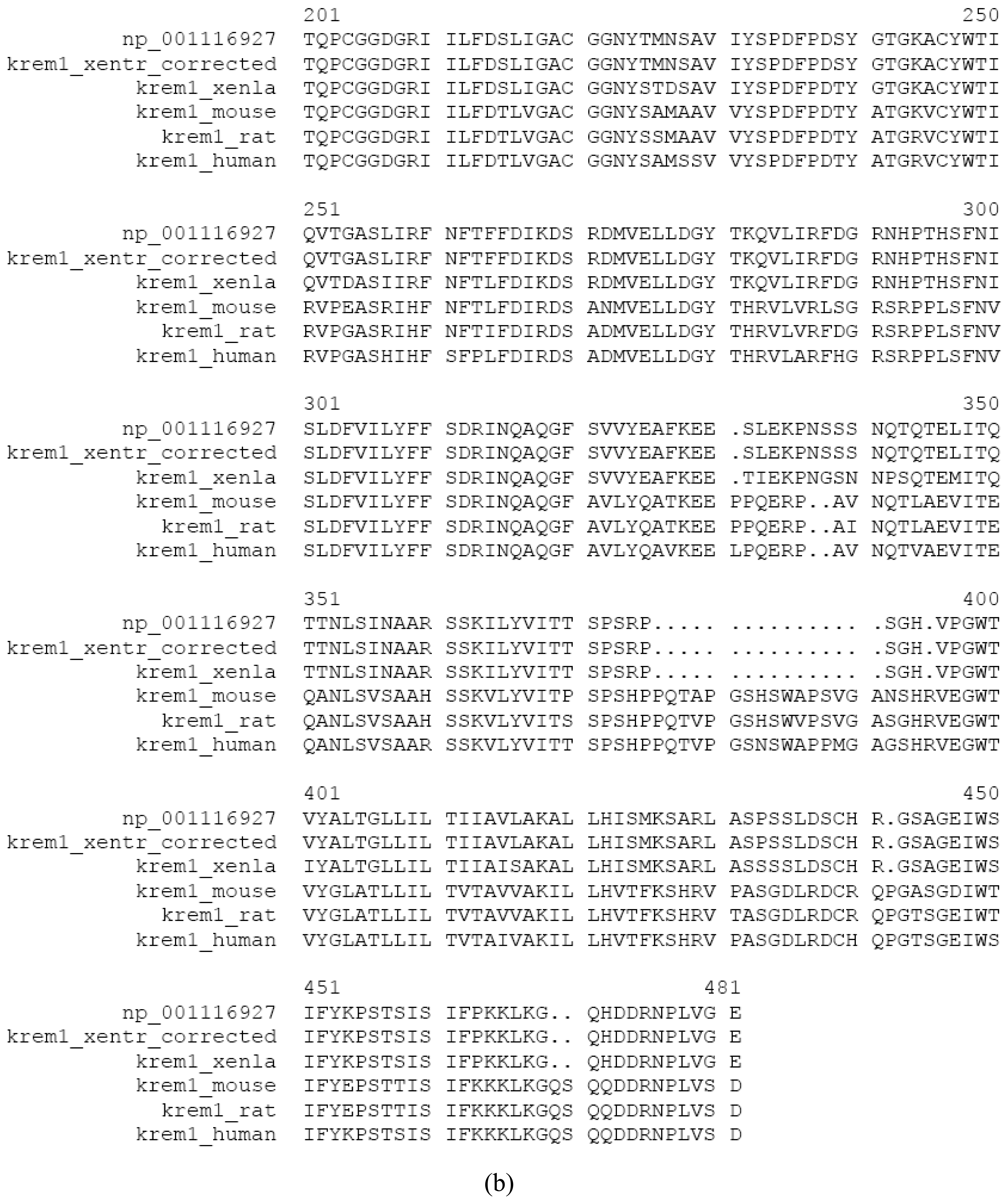

Supplement: Figure S6 — Correction of the sequence of the Kremen 1 protein of Xenopus tropicalis with the FixPred protocol. The DA of the Refseq ortholog of kremen 1 from Xenopus tropicalis (NP_001116927) was found to differ from that of KREM1_HUMAN: whereas the latter contains a Kringle, a WSC and a CUB domain (as well as a signal peptide and a transmembrane segment), the ortholog of Xenopus tropicalis lacks the kringle domain. The sequence ‘KREM1_XENTR_CORRECTED’ was predicted by the use of alternative gene models and is supported by ESTs DT392278 and EL798390. (a) Comparison of the DAs of KREM1_HUMAN, NP_001116927 and KREM1_XENTR_CORRECTED; (b) Alignment of the sequences of KREM1 proteins from human, mouse, rat and the frog Xenopus laevis with NP_001116927 and the corrected sequence, KREM1_XENTR_CORRECTED. [file genes-02-00449f8b.tif]

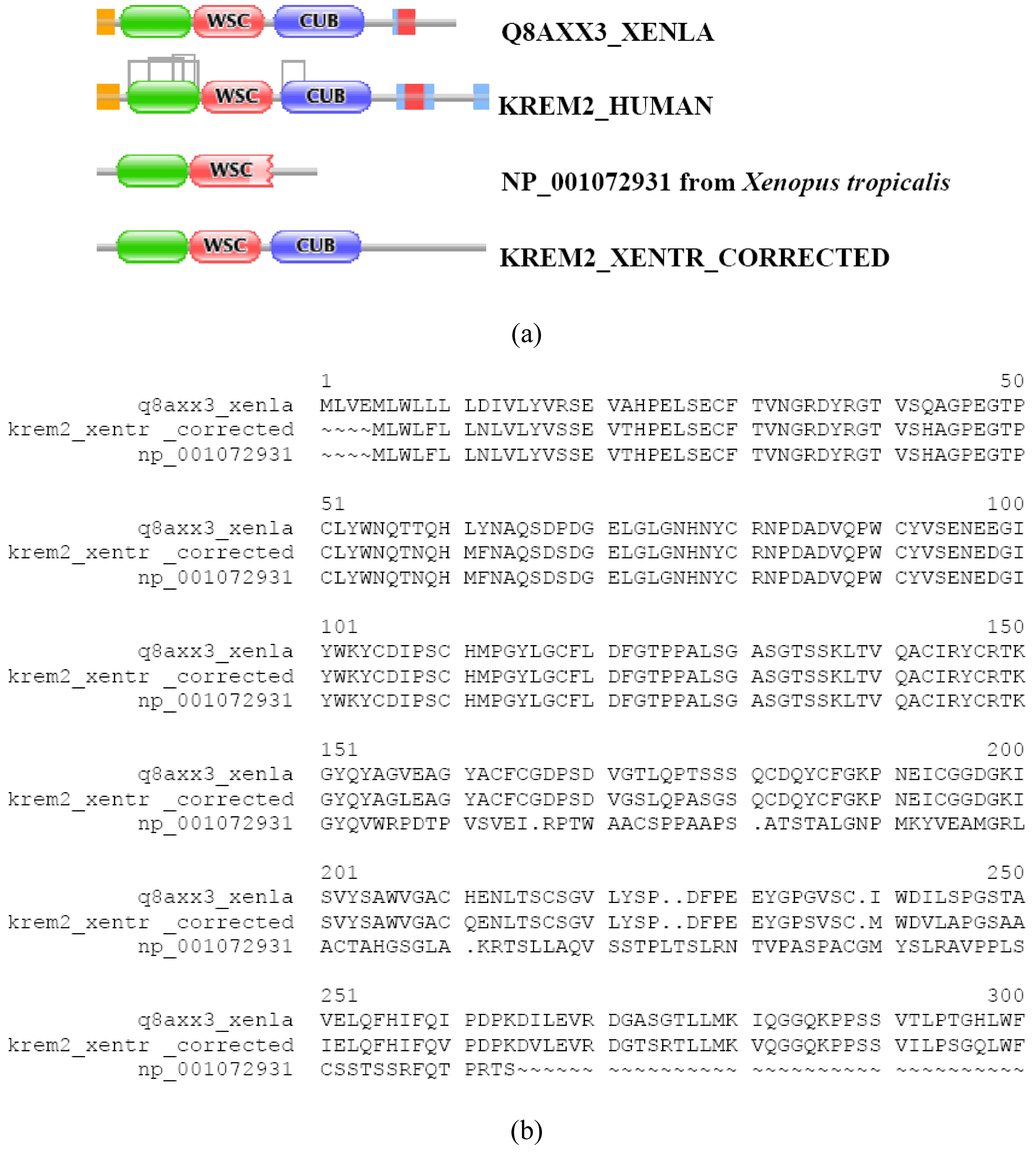

Supplement: Figure S7 — Correction of the sequence of the Kremen 2 protein of Xenopus tropicalis with the FixPred protocol. The DA of Refseq ortholog of kremen 2 from Xenopus tropicalis (NP_001072931) was found to differ from those of KREM1_HUMAN, KREM1_MOUSE, KREM1_RAT and the ortholog from Xenopus laevis, Q8AXX3_XENLA: whereas the latter contain a Kringle, a WSC and a CUB domain (as well as a signal peptide and a transmembrane segment), the ortholog of Xenopus tropicalis lacks the CUB domain. The sequence ‘KREM2_XENTR_CORRECTED’ was predicted by the use of alternative gene models and is supported by ESTs DT425049 and DT425818. (a) Comparison of the DAs of KREM2_HUMAN, Q8AXX3_XENLA, NP_001072931 and KREM1_XENTR_CORRECTED; (b) Alignment of the sequence of Q8AXX3_XENLA with NP_001072931 and the corrected sequence, KREM1_XENTR_CORRECTED. [file genes-02-00449f9a.tif]

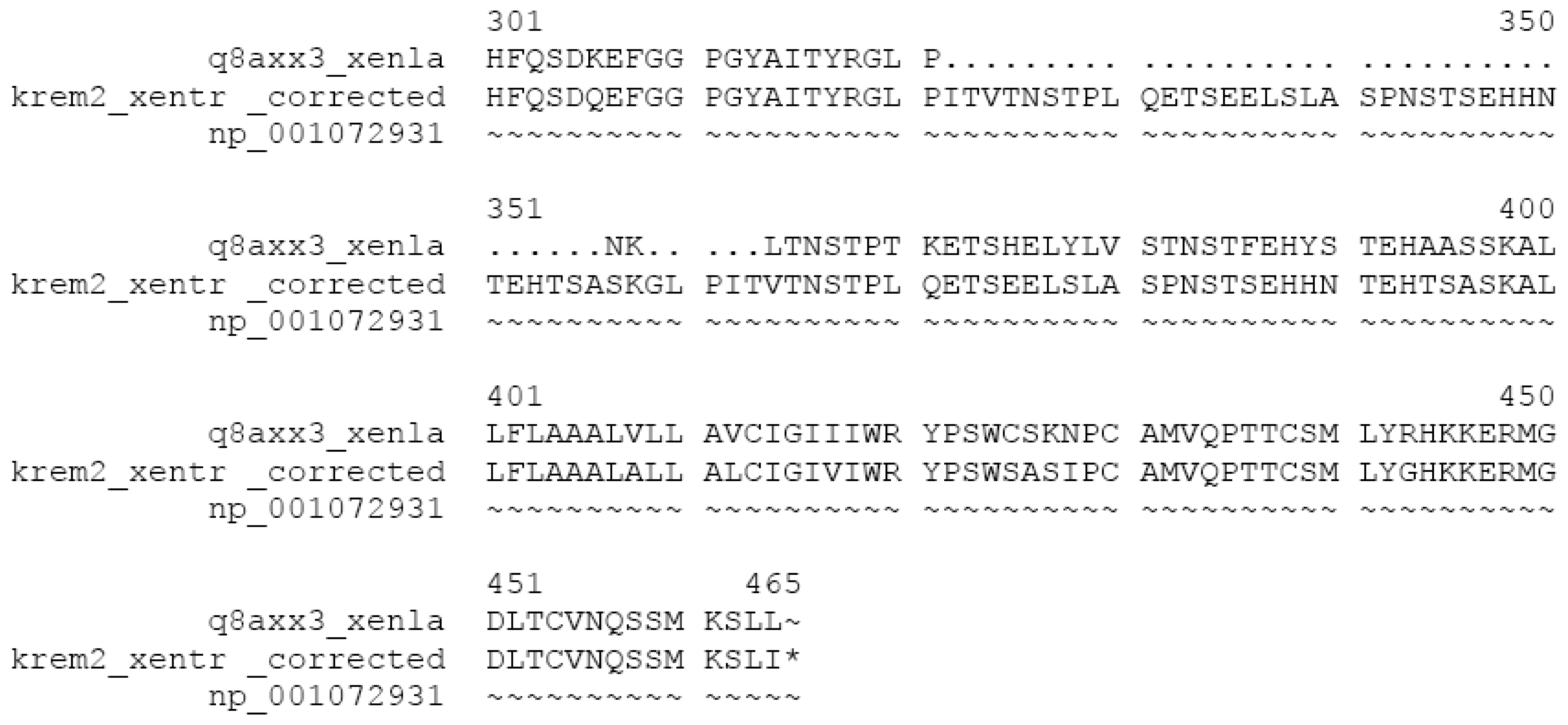

Supplement: Figure S7 — Correction of the sequence of the Kremen 2 protein of Xenopus tropicalis with the FixPred protocol. The DA of Refseq ortholog of kremen 2 from Xenopus tropicalis (NP_001072931) was found to differ from those of KREM1_HUMAN, KREM1_MOUSE, KREM1_RAT and the ortholog from Xenopus laevis, Q8AXX3_XENLA: whereas the latter contain a Kringle, a WSC and a CUB domain (as well as a signal peptide and a transmembrane segment), the ortholog of Xenopus tropicalis lacks the CUB domain. The sequence ‘KREM2_XENTR_CORRECTED’ was predicted by the use of alternative gene models and is supported by ESTs DT425049 and DT425818. (a) Comparison of the DAs of KREM2_HUMAN, Q8AXX3_XENLA, NP_001072931 and KREM1_XENTR_CORRECTED; (b) Alignment of the sequence of Q8AXX3_XENLA with NP_001072931 and the corrected sequence, KREM1_XENTR_CORRECTED. [file genes-02-00449f9b.tif]

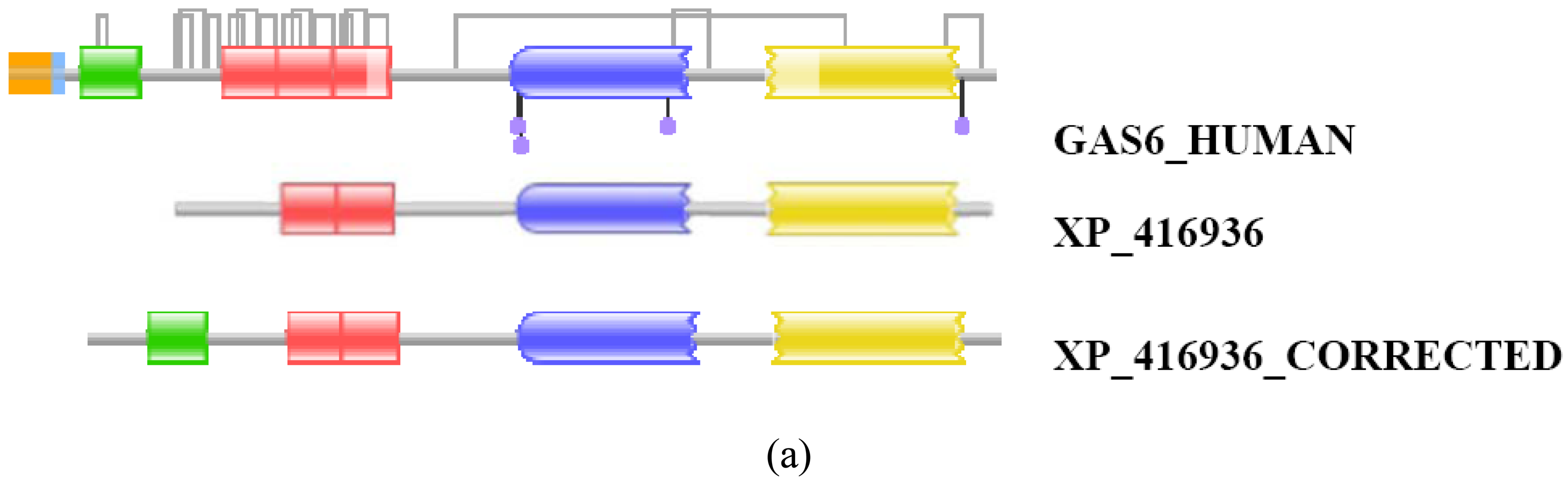

Supplement: Figure S8 — Correction of the sequence of the XP_416936 protein of Gallus gallus with the FixPred protocol. The DA of the GNOMON predicted protein XP_416936 was found to differ from those of GAS6_MOUSE, GAS6_RAT, GAS6_HUMAN: whereas the latter contain a signal peptide, a Gla, three EGF_CA, a Laminin_G_1 and a Laminin_G_2 domain, XP_416936 lacks the N-terminal signal peptide and Gal domain. The sequence XP_416936_CORRECTED was predicted by the use of ESTs CD217792, BM439645 and BU115578. (a) Comparison of the DAs of XP_416936, some of the four EGF_CA domains of GAS6 proteins are detected with E-values >0.0001 and are not represented in the DA images generated by Pfam. (b) Alignment of the sequences of XP_416936, XP_416936_CORRECTED with those of GAS6_MOUSE, GAS6_RAT and GAS6_HUMAN. Note that XP_416936_CORRECTED with those of GAS6_MOUSE, GAS6_RAT and GAS6_HUMAN. [file genes-02-00449f10a.tif]

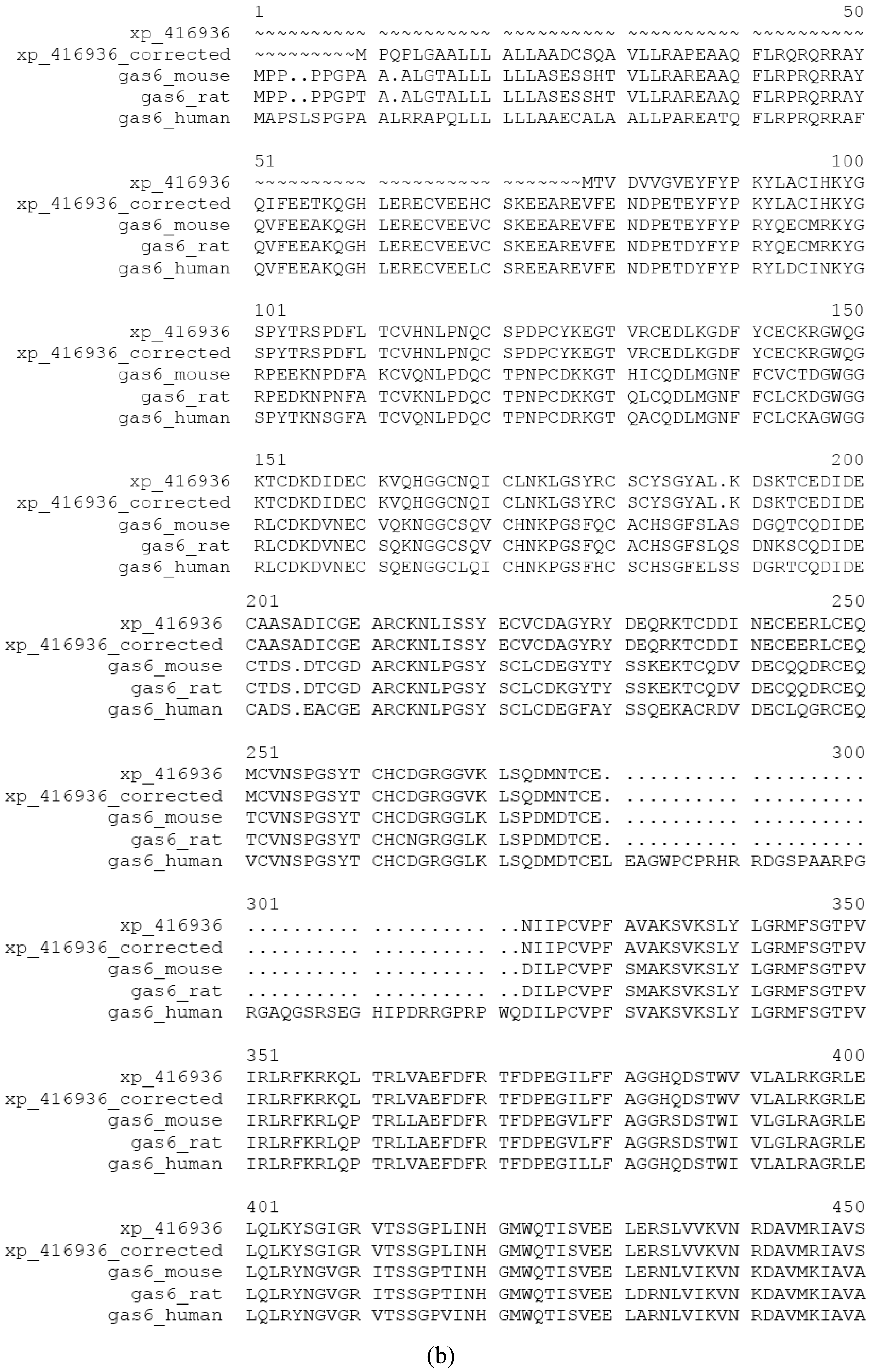

Supplement: Figure S8 — Correction of the sequence of the XP_416936 protein of Gallus gallus with the FixPred protocol. The DA of the GNOMON predicted protein XP_416936 was found to differ from those of GAS6_MOUSE, GAS6_RAT, GAS6_HUMAN: whereas the latter contain a signal peptide, a Gla, three EGF_CA, a Laminin_G_1 and a Laminin_G_2 domain, XP_416936 lacks the N-terminal signal peptide and Gal domain. The sequence XP_416936_CORRECTED was predicted by the use of ESTs CD217792, BM439645 and BU115578. (a) Comparison of the DAs of XP_416936, some of the four EGF_CA domains of GAS6 proteins are detected with E-values >0.0001 and are not represented in the DA images generated by Pfam. (b) Alignment of the sequences of XP_416936, XP_416936_CORRECTED with those of GAS6_MOUSE, GAS6_RAT and GAS6_HUMAN. Note that XP_416936_CORRECTED with those of GAS6_MOUSE, GAS6_RAT and GAS6_HUMAN. [file genes-02-00449f10b.tif]

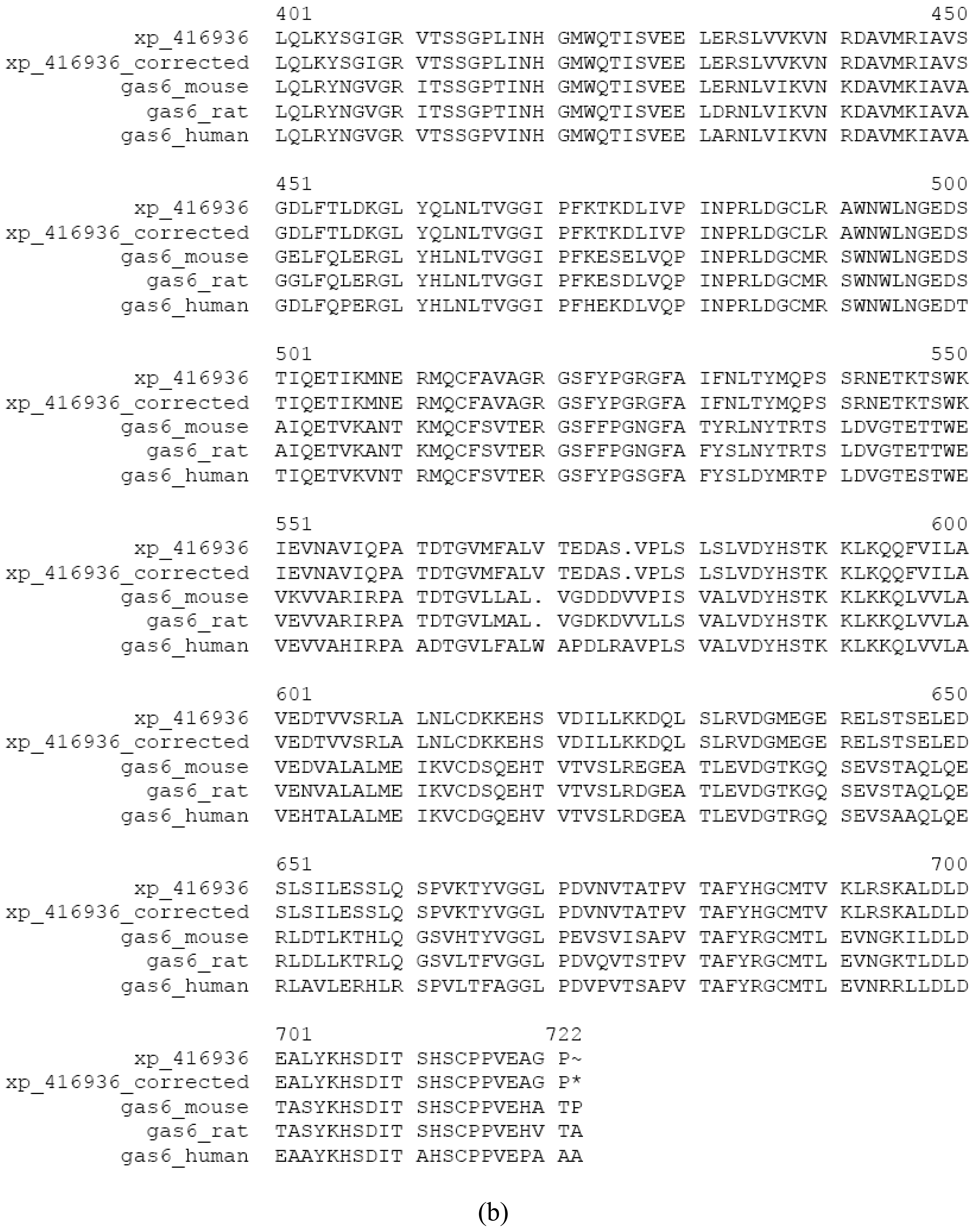

Supplement: Figure S8 — Correction of the sequence of the XP_416936 protein of Gallus gallus with the FixPred protocol. The DA of the GNOMON predicted protein XP_416936 was found to differ from those of GAS6_MOUSE, GAS6_RAT, GAS6_HUMAN: whereas the latter contain a signal peptide, a Gla, three EGF_CA, a Laminin_G_1 and a Laminin_G_2 domain, XP_416936 lacks the N-terminal signal peptide and Gal domain. The sequence XP_416936_CORRECTED was predicted by the use of ESTs CD217792, BM439645 and BU115578. (a) Comparison of the DAs of XP_416936, some of the four EGF_CA domains of GAS6 proteins are detected with E-values >0.0001 and are not represented in the DA images generated by Pfam. (b) Alignment of the sequences of XP_416936, XP_416936_CORRECTED with those of GAS6_MOUSE, GAS6_RAT and GAS6_HUMAN. Note that XP_416936_CORRECTED with those of GAS6_MOUSE, GAS6_RAT and GAS6_HUMAN. [file genes-02-00449f10c.tif]
